# Metabolomic and Transcriptomic Analysis of Flavonoid Biosynthesis in Two Main Cultivars of *Actinidia arguta* Sieb.Zucc. Grown in Northern China

**DOI:** 10.3389/fpls.2022.911203

**Published:** 2022-06-30

**Authors:** Yubo Wang, Yong Wang, Jun Sun, Yue Dai, Fengyan Yang, Hui Jiang, Muhammad Irfan, Lijing Chen

**Affiliations:** ^1^Key Laboratory of Agriculture Biotechnology, College of Biosciences and Biotechnology, Shenyang Agricultural University, Shenyang, China; ^2^College of Chemical Engineering, University of Science and Technology Liaoning, Anshan, China; ^3^Liaoning Institute of Economic Forestry, Dalian, China; ^4^Shandong Xianda Agrochemical Co., Ltd, Jinan, China; ^5^Shenyang Modern Agricultural R&D Service Center, Shenyang Academy of Agricultural Sciences, Shenyang, China; ^6^Department of Biotechnology, University of Sargodha, Sargodha, Pakistan

**Keywords:** *Actinidia arguta* Sieb.Zucc., flavonoids, biosynthesis, widely targeted metabolome, transcriptome, joint analysis

## Abstract

*Actinidia arguta* Sieb.Zucc. is a fruit that is rich in flavonoids. Nevertheless, details of flavonoid formation and the potential mechanism behind flavonoid biosynthesis have not previously been reported. In order to explore the biosynthetic regulation mechanism of flavonoids in *A. arguta* Sieb.Zucc., we conducted a combination of extensive targeted metabolite analysis and analyzed transcriptomes to determine the flavonoids present and the genes bound up with flavonoid biosynthesis in the two main cultivated varieties of *A. arguta* Sieb.Zucc. in Northern China. The maturity period is from August to September. A total of 118 flavonoids were found in fruits. Among them, 39 flavonoids were accumulated at significant levels after fruit ripening. Transcriptome analysis indicated that most flavonoid biosynthesis structural genes and certain regulatory genes exhibited differential expression between the two varieties. Correlation analysis of transcriptome and metabolite profiles showed that the ways of expression of 21 differentially expressed genes related to structure and regulation between the 2 varieties were more highly correlated with 7 flavonoids after fruit ripening. These results contribute to the development of *A. arguta* Sieb.Zucc. as a food and drug homologous functional food.

## Key Message

Transcriptome and widely targeted metabolomics were used to analyze the biosynthesis mechanism of flavonoids in *Actinidia arguta* Sieb.Zucc., so as to provide valuable information for its development as a food and drug homologous functional food.

## Introduction

*Actinidia arguta* Sieb.Zucc. commonly known as soft jujube kiwifruit, macaque pear, or rattan melon, is a perennial plant in the Actinidiaceae family. It is distributed in Jilin, Heilongjiang, Liaoning, and Shandong Provinces in North and Northwest China, and matures from June to August each year. It’s one of the famous and economically important wild fruits in the Changbai Mountain Area of China. Due to its green color, juiciness, and delicately sweet and sour flavor, it is known as “the precious fruit of the world” (Zuo et al., 2012; Hãdãrugã et al., 2016). *A. arguta* Sieb.Zucc. is nutritionally rich and contains a variety of functional components, such as amino acids, vitamins, and flavonoids. It is an excellent raw material for the growth of functional health foods (Latocha et al., 2014; Pal et al., 2015; Almeida et al., 2018). Flavonoids are one of the main active components of *A. arguta* Sieb.Zucc. grown in Changbai Mountain. They have anti-oxidation and anti-viral functions. In addition to preventing cardiovascular disease, cerebrovascular disease, and hyperuricemia, they contribute to liver protection and immunity (Latocha et al., 2013; Hu et al., 2016; Jiang et al., 2020). *A. arguta* Sieb.Zucc. has broad application prospects for the development of flavonoid products in the field of medicine and food homology. However, the key compounds present in the fruit have not been systematically analyzed.

Thus far, 10 flavonoids have been detected in *A. arguta* Sieb.Zucc. leaves, including quercetin, isorhamnetin, and kaempferol. Furthermore, a total of 70,631 unigenes have been identified in this plant from transcriptome data, which, in fruits and leaves, includes 29,617 up-regulated and 2976 down-regulated genes. Key genes, including *AaF3′5′H-1H* and *AaF3H-1*, have higher levels of expression in the leaves than in other tissues (Tan et al., 2021). The fruits of two *A. arguta* Sieb.Zucc. varieties were RNA sequenced (RNA-Seq), and indicated that leucoanthocyanidin dioxygenase (LDOX) may be the key gene regulating anthocyanin synthesis in kiwifruit pulp of the red-fruit variety “tianyuanhong” (Li et al., 2018). In addition, comparing homologous genes in *Arabidopsis thaliana* indicated that the majority of structural genes bound up with flavonoid biosynthesis in bitter mustard have been appraised, and the functions of some have been verified (Gupta et al., 2011; Park et al., 2011; Li C. et al., 2012; Li C. L. et al., 2012; Li X. et al., 2012; Yao et al., 2016; Zhang et al., 2017). FtMYB1 and FtMYB2 belong to the R2R3 MYB family and share high homology with AtMYB123/TT2. They actively mediate proanthocyanidin accumulation in transgenic tobacco by affecting the expression of genes bound up with flavonoid structure, including PAL, CHI, FLS, F3H, DFR, and ANS (Bai et al., 2014). Similarly, FtMYB15 and FtWD10 also have a positive effect on anthocyanin and procyanidin biosynthesis by enhancing the expression of flavonoid biosynthesis genes in early and late tobacco and tartary buckwheat (Yao et al., 2017; Luo et al., 2018). In addition, the transcription factor FtMYB116, which is induced by blue and red light, promotes rutin accumulation in light-induced bitter mustard *via* the direct regulation of F3′H expression and the indirect regulation of PAL, CHS, FLS, and F3H expression (Zhang et al., 2019).

More than a dozen flavonoids and structural genes have been identified in the leaves of *A. arguta* Sieb.Zucc. However, there have been no reports of systematically studying the composition of flavonoids in *A. arguta* Sieb.Zucc. and analyzing the molecular mechanisms of flavonoid biosynthesis. Here, we performed extensive transcriptomic and targeted metabolite analysis of flavonoids in the mature fruits of two important *A. arguta* Sieb.Zucc. cultivars grown in Northern China. A total of 118 flavonoids were identified, 39 of which showed different accumulation patterns between the 2 varieties. In addition, dozens of structural and transcriptional genes related to the regulation of flavonoid biosynthesis were found DEGs. Among them, the expression patterns of 21 were more strongly correlated with 7 flavonoids between the 2 varieties after fruit ripening. Certain DEG expression levels were validated using qRT-PCR. This data contributes to a better understanding of flavonoid biosynthesis in *A. arguta* Sieb.Zucc. at both the molecular and metabolic levels and provides precious information for future fruit quality improvement, cultivation of novel strains, and development of functional foods.

## Materials and Methods

### Plant Materials and Sampling

Mature fruit which have a fruit maturity from August to September was collected from 8-year-old *A. arguta* Sieb.Zucc. varieties (Qssg and Lc) grown in Northeast, North, and Northwest China and Shandong Province. There were three independent biological replicates for each sample. Collect the fresh fruit samples and put them in liquid nitrogen.

### Composition Analysis of Flavonoids in Two Kinds of *Actinidia arguta* Sieb.Zucc.

Total flavonoids were measured in the fruit samples. Metware Biotechnology Co., Ltd. (Wuhan, China) conducted 785 metabolite analyses. In short, cryopreserved samples at ultra-low temperatures were freeze-dried and ground with a grinder until it was a powder (Mixer Mill MM 400, Retsch, Haan, Germany) at 30 Hz for 1.5 min. Next, 100 mg of powder was mixed with 1.2 ml of 70% methanol. Samples were vortexed once every 30 min for 30 s a total of six times, then incubated overnight at 4°C. Retain the filtrate with a microporous membrane. A UPLC-MS/MS system was used to analyze the samples. Flavonoids were identified using a local database. A multiple reaction monitoring (MRM) model was used to quantitatively analyze the flavonoids (Chen et al., 2013; Dong et al., 2014, 2019). Flavonoids were further annotated online with PlantCyc^1^ and the KEGG.^2^ Principal component analysis (PCA) and orthogonal partial least squares discriminant analysis (OPLS-DA) were used to determine differentially accumulated flavonoids, where |log2 (fold change)| ≥ 1 was used as the threshold.

### RNA Extraction, Library Construction, and Sequencing

An EasySpin Plus Plant RNA Kit (Aidlab) was used to obtain total RNA from frozen fruits, which was then treated with DNase I (Takara) to clear it of contaminating DNA. mRNA was cleaned from the total RNA with a Dynabeads mRNA Purification Kit (Invitrogen, Waltham, MA, United States). The resulting purified mRNA was fragmented, after which first-strand cDNA was synthesized using random hexamer primers, and second-strand cDNA was synthesized with a NEBNext Ultra RNA Library Preparation Kit (New England Biolabs, Ipswich, MA, United States). cDNA was purified, ends were repaired, and the fragments were ligated to adapters. cDNA was then fragmented again after which it was enriched *via* PCR and the last cDNA library was built for Illumina paired-end sequencing. Biomarker Technology Co., Ltd. (Beijing, China) sequenced the cDNA library using the HiSeq Xten PE150 platform (Illumina, San Diego, CA, United States).

### Data Analysis

#### Metabolomic Data Analysis

Based on MVDB V2.0 built by Maiwei (Wuhan) Biotechnology Co., Ltd. database and metabolite information public database. Qualitative analysis is carried out according to the secondary spectrum information. The quantitative analysis of metabolites is completed by triple quadrupole mass spectrometry multi reaction monitoring mode (MRM). The metabolic material spectra of different samples are obtained, and the mass spectrum peaks of substances are integrated by peak area. The mass spectrum peaks of the same metabolite in different samples are integrated and corrected, and the mass spectrum data are processed by Software Analyst 1.63. Multivariate statistical analysis was used to conduct PCA and cluster analysis on the two groups of samples. The stability and reliability of the model were predicted according to partial least squares discriminant analysis (PLS-DA) and OPLS-DA. Multi-dimensional statistical variable importance in the project (VIP) value, one-dimensional statistical *p*-value and differential multiple were used to screen differential metabolites. Through the pheatmap program in R (v3.2), the differential metabolic components in the fruit of *A. arguta* Sieb.Zucc. were clustered and analyzed, and the heat map was drawn. The differential metabolites in each group were screened by hierarchical clustering, and the corresponding differential metabolites were submitted to KEGG website for related pathway analysis.

#### Transcriptome Data Analysis

Fastp software is used to strictly control the quality of RNA data and the adapter sequences and low-quality reads were removed to obtain clean reads, which we then mapped to the ACTIN reference gene sequence.^3^ Unigenes were determined using hisat2 and stringtie. Gene functional annotation was performed using BLAST to search several databases, namely the NCBI NR database, Swiss-Prot, GO, COG, KOG, Pfam, and KEGG, using an *e*-value cutoff of 10^–5^. The expression level for each gene was determined and then normalized according to the number of FPKM. The deseq software package was used to identify DEGs between samples. FDR < 0.05 and |log2 (fold change)| ≥ 1 were used as thresholds for defining DEGs. Functional enrichment analysis was performed on the DEGs using GO annotations and KEGG pathways.

#### qRT-PCR Analysis

qRT-PCR was used to validate the RNA-Seq data and measure the expression of structural genes involved in flavonoid biosynthesis. qRT-PCR analysis was conducted according to previously outlined methods (Park et al., 2011; Tan et al., 2021). The amplification cycle procedure is as follows: the reverse transcription operation is carried out with the reverse transcription kit (TUREscript 1st Stand cDNA SYNTHESIS Kit) of Aidlab Company. A total 20 μl reaction system is adopted and the reverse transcription reaction conditions were 42°C for 40 min and 65°C for 10 min. Fluorescence quantitative PCR procedure and system: 95°C 3 min, 95°C 10 s, 60°C 30 s. Taking ACTIN as the internal reference, the gene expression data are presented as relative expression, the relative expression of genes in each sample and each group was calculated by 2^–ΔΔ*Ct*^. Three biological replicates were performed on each sample. Supplementary Table 1 lists the primers used in qRT-PCR.

### Correlation Analysis Between Metabolite Spectrum and Transcripts

The correlation analysis was promoted by identifying the correlation coefficient of the content of flavonoids and the transcriptional changes in both differentially expressed flavonoids and differentially expressed genes. Both of them are rich in flavonoids and flavonol biosynthesis pathway (ko00941), flavonoid biosynthesis pathway (ko00942), and secondary metabolite biosynthesis pathway (ko00943). The interaction network between DEGs and differentially accumulated flavonoids was visualized with Cytoscape2.8.

## Results

### Flavonoid Composition in Two *Actinidia arguta* Sieb.Zucc. Varieties

**FIGURE 1:**
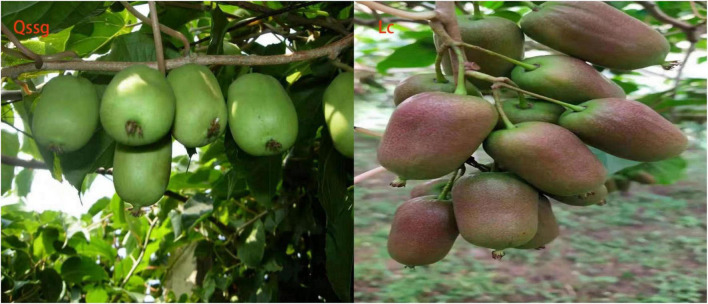
Qssg and Lc of two main cultivars of *Actinidia arguta* Sieb.Zucc. in Northern China.

To study the composition of flavonoids during the fruit ripening stage of *A. arguta* Sieb.Zucc. varieties Qssg and Lc (Figure 1), widely targeted metabonomics was used for the first time. LC-ESI-QTrap-MS/MS and multiple reaction monitoring were used to generate multi-peak maps. Specific ions for each substance were identified with the triple four-stage rod, while the signal strength (CPS) of the characteristic ions was generated using a detector. We opened the sample off-line mass spectrometry file using the multiquant software to conduct qualitative and quantitative analyses of the metabolite spectrum in Qssg and Lc *A. arguta* Sieb.Zucc.

**FIGURE 2:**
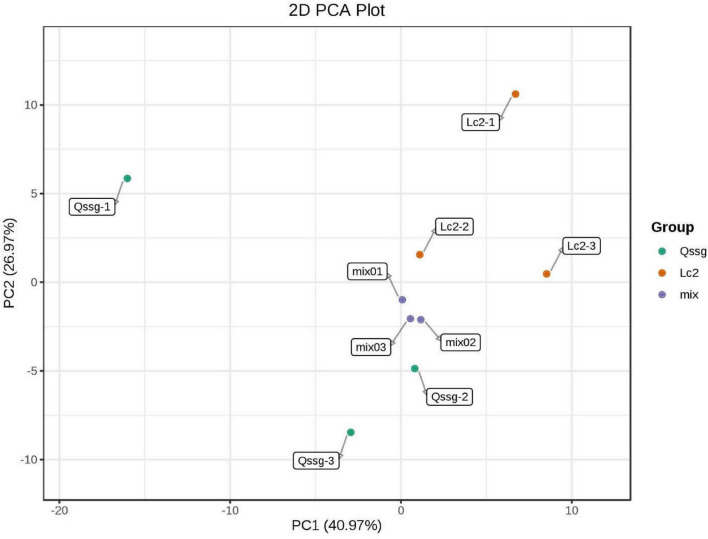
Principal component analysis score map of metabolites in Qssg and Lc fruits. Each point represents an independent biological repeat.

**FIGURE 3:**
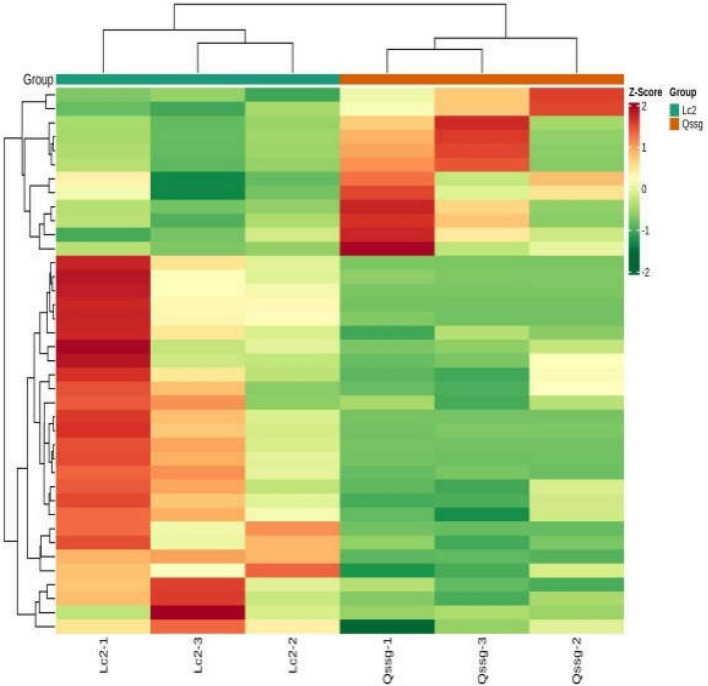
Cluster heat map of metabolites in Qssg and Lc fruits. There are obvious differences between the two samples.

A total of 118 flavonoids were identified, including 18 flavone, 6 dihydroflavonols, 12 Dihydroflavones, 12 flavonols, 64 flavonols, 2 chalcone compounds, 3 flavonoid carboglycosides, and 1 isoflavone (Supplementary Table 2). PCA highlighted that these two varieties were significantly different; 67.94% of differences between the samples could be explained by PC1 (40.97%) and PC2 (26.97%), indicating a change in the pattern of flavonoid accumulation between the two varieties (Figure 2). Hierarchical cluster analysis (HCA) based on the relative content of flavonoids in different cultivated varieties of *A. arguta* Sieb.Zucc. during fruit ripening showed that the concentration of most detected flavonoids changed greatly during fruit ripening. Among the 39 different flavonoids, comparing Qssg with Lc, 12 flavonoids in the fruit were up-regulated, that is, the relative content increased, and the increased metabolic components accounted for 30.8% of the 39 different metabolic components; 27 components were down regulated, that is, the relative content decreased, and the reduced metabolic components accounted for 69.2% of the 39 differential metabolic components. This further confirmed the difference in PCA results between the two main samples (Figure 3).

### Differences in Flavonoids Between Two *Actinidia arguta* Sieb.Zucc. Varieties

#### Results of Orthogonal Partial Least Squares Discriminant Analysis

**FIGURE 4:**
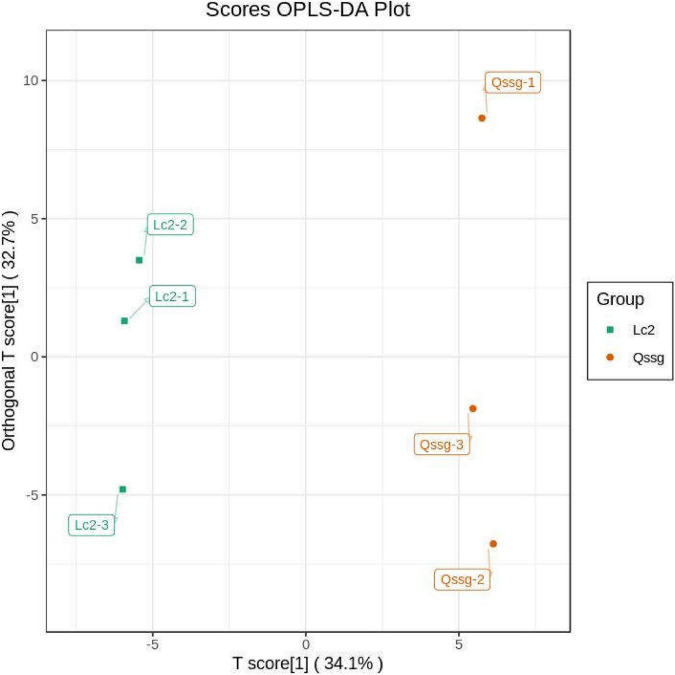
Orthogonal partial least squares discriminant analysis score map of metabolites in Qssg and Lc fruits. Each point represents an independent biological repeat.

Orthogonal partial least squares discriminant analysis can maximize the distinction among categories and identify differentially accumulated metabolites. It uses OSC and PLS-DA techniques to identify variable differences. The abundance of the 118 flavonoids was compared between samples using the OPLS-DA model (Figure 4). Lc samples were found to the left of the confidence interval, while the Qssg samples were found to the right of the confidence interval, clearly demonstrating the ability of the model to discriminate between the two varieties.

**FIGURE 5:**
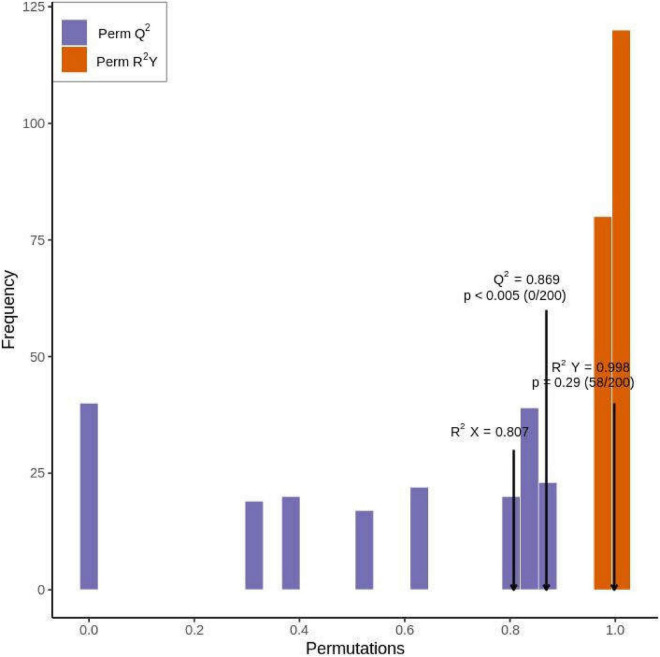
Orthogonal partial least squares discriminant analysis model validation diagram of metabolites in Qssg and Lc fruits (*p* < 0.005; *n* = 200, that is, 200 alignment experiments were carried out).

We generated two principal components from OPLS-DA; the contribution rate was 62.1% for principal component 1 and 9.74% for principal component 2 [r2x = 0.807, r2y = 0.998 (*p* = 0.29), Q2 = 0.869 (*p* < 0.005)]. The OPLS-DA results were verified by performing 200 replicates of the analysis. Model verification (Figure 5) proved that the OPLS-DA model was biologically meaningful and superior to the PCA model.

#### Identification of Differentially Accumulated Flavonoids

The OPLS-DA results (Figure 5), the multivariable analysis, and the VIP value of VIP of the OPLS-DA model represent the influence intensity of the intergroup difference of the related metabolites when classifying and discriminating samples in models from each group. It is generally considered that metabolites with VIP ≥1 have a significant difference. Three classes and 39 species of differentially accumulated flavonoids were identified (Supplementary Table 3), accounting for 33.1% of all identified flavonoids (118). Specifically, 56.4% of the differentially accumulated flavonoids were flavonols, 10.3% were flavones, 10.3% were dihydroflavonoids, 10.3% were flavanols, 5.1% were chalcones, 5.1% were flavone carboglycosides, and 2.5% were dihydroflavonols. This suggests the presence of significant differences between flavonoids between different varieties of *A. arguta* Sieb.Zucc.

#### Thermogram Analysis of Differentially Accumulated Flavonoids

**FIGURE 6:**
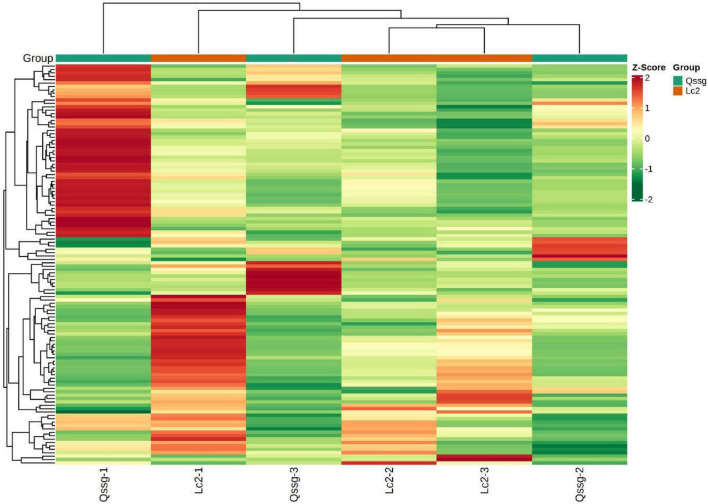
Clustering heat map of flavonoids with significant difference between Qssg and Lc fruits.

To facilitate observation of the change law of flavonoids, significantly different flavonoids were normalized, and a clustering heat map was generated (Figure 6).

#### Analysis of Key Differentially Accumulated Flavonoids

**FIGURE 7:**
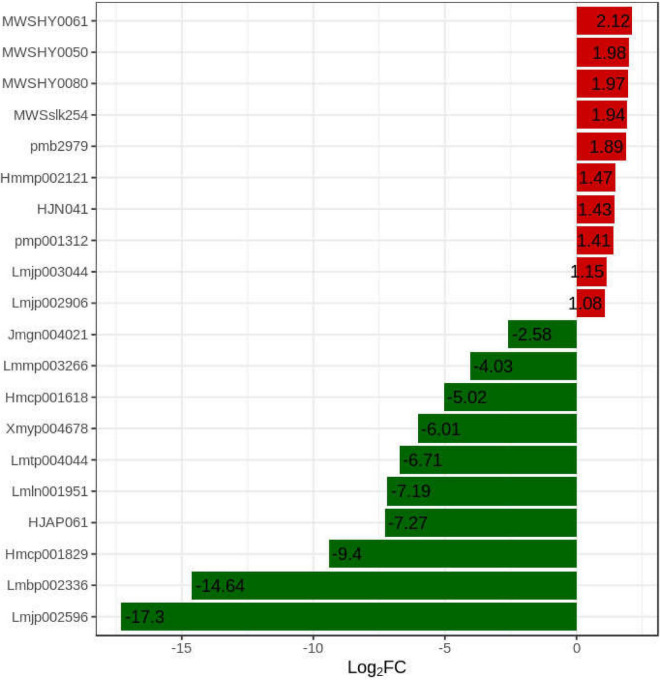
Difference multiples of flavonoids in Qssg and Lc fruits variation diagram. (The abscissa is the difference multiple of differential metabolites, with 2 as the base pair, and the ordinate is the differential metabolite. Red and green represent up regulation and down regulation of differential metabolites, respectively).

Compare the difference multiple changes of quantitative information of flavonoids in two *A. arguta* Sieb.Zucc. samples of Qssg and Lc, and process the difference multiple with log2. The 20 most common differentially expressed flavonoids are shown (Figure 7). The relative flavonoid content of Lc variety fruits was significantly higher (1.34 times) than the Qssg fruits.

**FIGURE 8:**
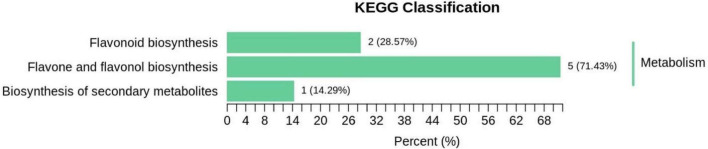
Classification of differential metabolites KEGG in Qssg and Lc fruits.

Pathway enrichment analysis of differentially accumulated flavonoids was conducted with KEGG (Figure 8). The 39 significantly different flavonoids identified were mainly distributed among 3 metabolic pathways: (1) flavonoid and flavonol biosynthesis pathways, primarily for kaempferol-3-o-neohesperidin Luteolin-7-o-neohesperidin, quercetin-3-o- sangbu diglycoside, quercetin-3-o-(2″-o-xylosyl)-rutoside, and kaempferol-3-o-rutoside; (2) flavonoid biosynthesis pathways, mainly including naringin-7-o-glucoside and isolyceride; and (3) secondary metabolite biosynthesis pathways, primarily kaempferol-3-o-rutoside.

#### Transcriptome Analysis of Two *Actinidia arguta* Sieb.Zucc. Varieties

To further study potential molecular mechanisms of flavonoid biosynthesis in *A. arguta* Sieb.Zucc., six cDNA libraries were constructed for high-throughput RNA-Seq analysis. Each library obtained 281,758,296 reads from 439,76,908 to 43,580,384, and each library obtained 269,732,354 clean reads from 41,844,326 to 41,708,692 (Supplementary Table 4). The q30 percentage (which includes sequences having an error rate < 0.1%) for each library exceeded 91%, and the mean GC content was 47.47%. A total of 78.54–80.76% of the clean reads were mapped to the transcripts of each library assembled and de redundant by Trinity, which were used as the reference sequence, and the clean reads of each sample were compared to the reference sequence. A total of 43,686 unique genes were identified. The analyses suggest high-quality RNA-Seq data suitable for use in further analyses.

#### Differential Gene Expression in Two *Actinidia arguta* Sieb.Zucc. Varieties

**FIGURE 9:**
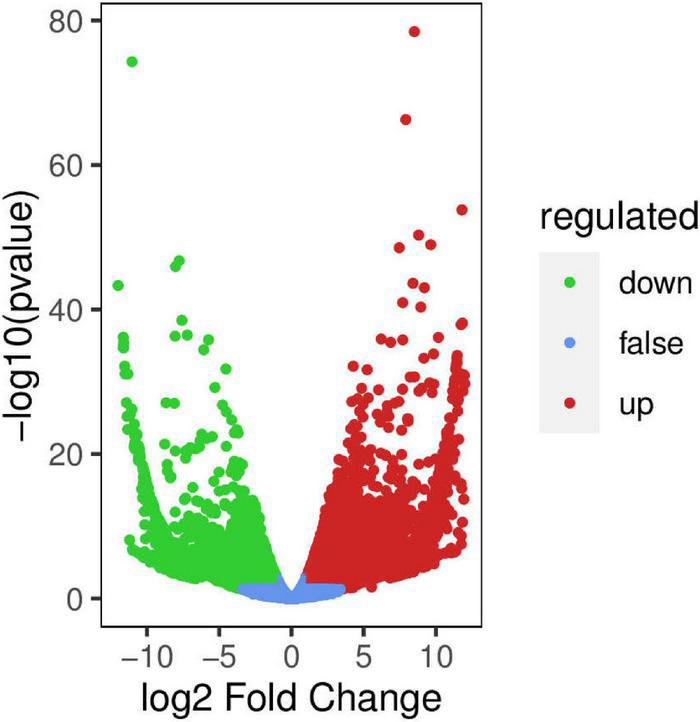
Volcano map of differential genes between Qssg and Lc fruits.

**FIGURE 10:**
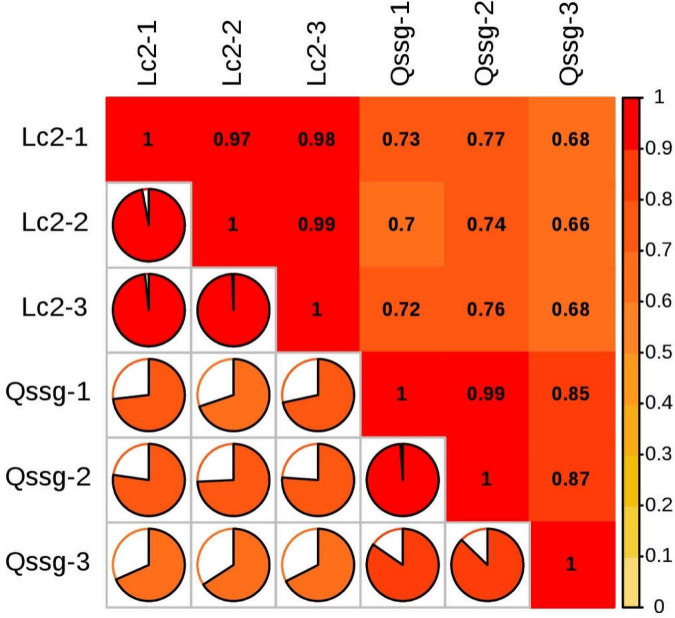
Heat map of gene correlation coefficient of difference in Qssg and Lc fruits.

For samples with biological duplication, DESeq2 was used for differential expression analysis between sample groups to obtain the differential expression gene set between the two biological conditions. The volcanic map can visually show the overall distribution of differential genes in the two groups of samples (Figure 9). DEGs in the mature fruits of two *A. arguta* Sieb.Zucc. varieties were identified by first analyzing the correlation coefficient between the profile clustering of gene expression and biological replicates. This analysis indicated that many genes exhibited differential expression in various samples. Correlation coefficients for the levels of gene expression of the biological replicates were >0.8 (Figure 10), indicating that the biological replicates were consistent with one another and the data were therefore valid for the identification of DEGs. Using threshold values of FDR < 0.05 and |log2 (fold change)| ≥ 1, DEGs between the 2 varieties were identified; there were 6497 up-regulated and 5153 down-regulated genes compared between Qssg and Lc.

**FIGURE 11:**
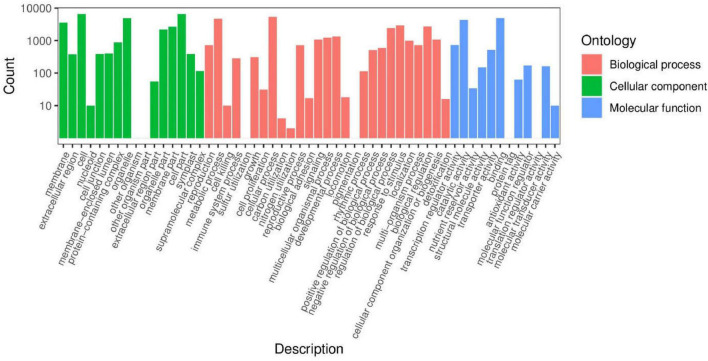
Classification of secondary entries of Qssg and Lc fruit difference genes.

To understand the biological functions of DEGs, we used Blast GO to determine enriched GO terms. Among the 55 enriched functional groups, there were 27 biological processes, 16 cell components, and 12 molecular functions (Figure 11 and Supplementary Table 5). Among the biological processes, the most frequent annotations were metabolic processes, cellular processes, and single biological processes. For cell component annotations, the most common terms were organelle, cell part, and cell. For molecular function annotations, the most represented terms were binding, catalytic activity, and transporter activity.

**FIGURE 12:**
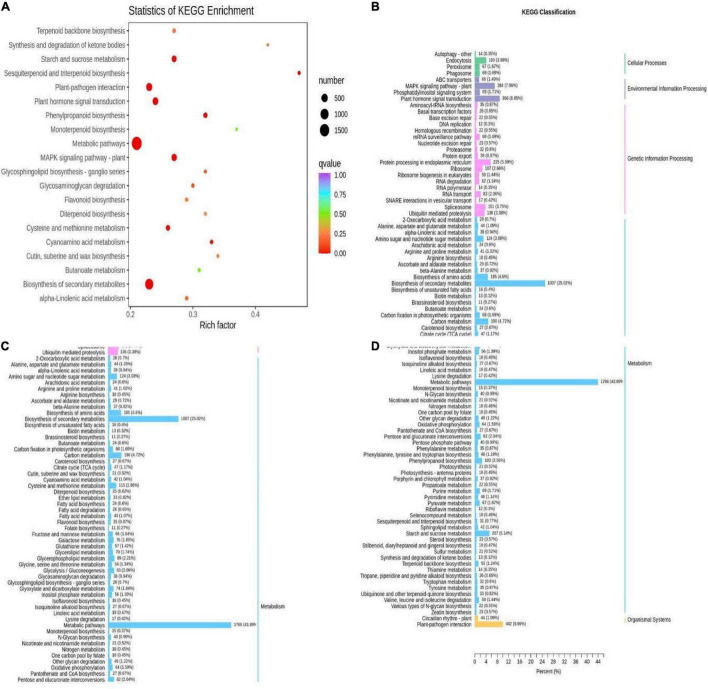
Enrichment pathways of different genes in Qssg and Lc fruits. **(A)** Scatter diagram of enrichment of differential gene KEGG in Qssg and Lc fruits. **(B–D)** Column diagram of enrichment of differential gene GO in Qssg and Lc.

KEGG analysis was conducted to identify enriched metabolic pathways in the DEGs. There were 4024 DEGs distributed among 142 KEGG pathways (Supplementary Table 6). Among them, there were 42 significantly enriched pathways (*p*-value < 0.05) (Supplementary Table 7). Flavonoid biosynthesis (ko00941), flavonoid and flavonol biosynthesis (ko00944), and secondary metabolite biosynthesis (ko01110) pathways were all enriched, and the flavonoid biosynthesis (ko00941) and secondary metabolite biosynthesis (ko01110) pathways were significantly enriched. Flavonoid biosynthesis (ko00941) and secondary metabolite biosynthesis (ko01110) exist in the pathway of significant enrichment (Figure 12A). All of the enriched pathways could be further divided into five categories: cellular processes, genetic information managing, environmental information, metabolism, and tissue systems. Among these categories, the metabolism category contained the most pathways, and the highest numbers of DEGs were involved in amino acid biosynthesis (ko01230; 185 genes), carbon metabolism (ko01200; 190 genes), and sucrose and starch metabolism (ko00500; 207 genes) (Figures 12B–D).

#### Analysis of Flavonoid Biosynthesis and Regulation of Differential Gene Expression in Two Kinds of *Actinidia arguta* Sieb.Zucc. at Maturity

The enriched KEGG pathways and gene functional annotations were used to determine which DEGs encode flavonoid biosynthesis-related enzymes, flavonoid, and flavonol biosynthesis-related enzymes, and secondary metabolite biosynthesis-related enzymes. In total, 41 were found, including 6 CsUGT134 genes, 17 LOC, 2 AT2, 1 CHS, 1 CHI, 3 C4H, 1HCT, 1 CCoAOMT, 1 F3H, 2 CFOL, 1 DFR, 3 LAR2, 1 LSAT, and 1 GSCOC (Supplementary Table 8). Among those, 6 UGT9491, 16 LOC, 2 AT2, 1 CHS, 2 C4H, 1 HCT, 1 CCoAOMT, 1 F3H, 2 LAR2, and 1 GSCOC were significantly up-regulated in the 2 varieties. A total of 2 CFOL, 1 CHI, 1 C4H, 1 DFR, 1 LAR2, 1 LOC, and 1 LSAT were significantly down-regulated between the two varieties.

#### qRT-PCR Confirmation of RNA Sequenced Results

**FIGURE 13:**
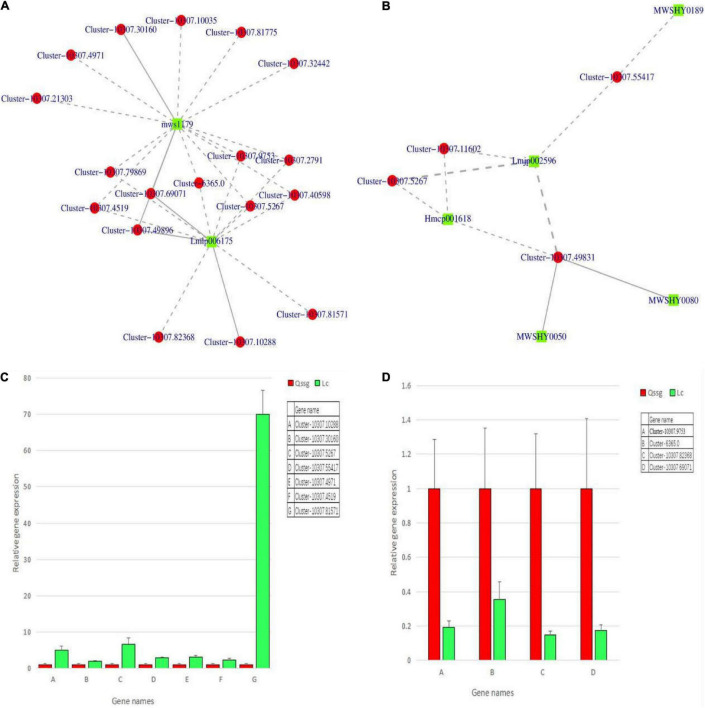
The differential flavonoid-related regulatory gene network in Qssg and Lc *Actinidia arguta* Sieb.Zucc. fruits and qRT-PCR confirmation map. **(A,B)** Twenty-one structural genes and 7 flavonoids showed higher correlation coefficient values (*r* > 0.8). **(C)** The relative expression of seven structural genes in Lc was higher than that in Qssg. **(D)** The relative expression of four structural genes in Lc was lower than that in Qssg. Most of the selected genes showed high correlation between the qPCR and RNA-Seq datasets, thus validating the transcriptome data (2^–ΔΔ*Ct*^ value of the internal reference gene is ACTIN).

To validate the expression of the DEGs related to flavonoid biosynthesis, qRT-PCR was performed for 11 structural genes (1 CsUGT134, 1 LAR2, 1 C4H, 1 CFOL, 1 CHI, 2 LOC, 1 GSCOC, 1 CCoAOMT, 1 DFR, and 1 AT2) in the fruits of the 2 varieties at maturity. The selected genes were highly correlated between RNA-Seq and qPCR data, effectively validating the transcriptome data (Figures 13C,D).

#### Correlation Between Transcripts and Flavonoid Derivatives

To better outline, the regulatory network controlling flavonoid biosynthesis, quantitative changes in flavonoids, and gene expression were compared. Based on the results of DEGs and DEMs rich in flavone biosynthesis pathway, 21 structural genes and 7 flavonoids (Supplementary Table 9) exhibited high correlation coefficients (*r* > 0.8). The interaction network is shown (Figures 13A,B). The 21 structural genes were as follows: 2 CsUGT134, 1 LAR2, 1 C4H, 2 CFOL, 1 CHI, 9 LOC, 1 GSCOC, 1 CCoAOMT, 1 DFR, and 2 AT2 genes. These are known to be the key structural genes related to the flavonoid biosynthesis pathway. The seven flavonoids were apigenin-7-o-glucoside, luteolin-7-o-neohesperidin (honeysuckle glycoside), kaempferol-3-o-rutoside (fireworks glycoside), quercetin-3-o-sangbu diglycoside, quercetin-3-o-(2″-o-xylosyl)-rutoside, naringin-7-o-glucoside (cherry glycoside), and isolyceride (phloridin chalcone). Eleven DEGs were further selected to verify the transcriptome results. qRT-PCR showed that the expression levels of C4H (Cluster-10307.4519), CsUGT134 (Cluster-10307.5267), AT2 (Cluster-10307.55417), LOC (Cluster-10307.81571, Cluster-10307.4971), DFR (Cluster-10307.30160), and CFOL (Cluster-10307.10288) in Lc were higher than those in Qssg. The expression levels of CHI (Cluster-10307.69071), LAR2 (Cluster-10307.9753), GSCOC (Cluster-6365.0), and CCOAOMT (Cluster-10307.82368) were inhibited in Lc. This means that these structural enzymes are required for flavonoid biosynthesis in *A. arguta* Sieb.Zucc. and the expression patterns among members may be opposite. It indicates that there are polygenic families, which may have different control mechanisms on the biosynthesis of flavonoids in *A. arguta* Sieb.Zucc. (Figure 13 and Supplementary Table 10).

## Discussion

*Actinidia arguta* Sieb.Zucc. is rich in bioactive flavonoids, which have been used to develop various health foods such as beverages, dried fruits, and wine. However, the flavonoid composition and potential mechanism regulating flavonoid biosynthesis in *A. arguta* Sieb.Zucc. remains largely unclear. Now, we conducted a comprehensive transcriptomic and metabolite analysis to determine the flavonoid composition in *A. arguta* Sieb.Zucc. and identify genes related to flavonoid biosynthesis.

We analyzed the metabolites to determine 118 flavonoids, greatly broadening our knowledge of flavonoids in the mature fruits of the 2 main varieties of *A. arguta* Sieb.Zucc. grown in Northern China. Among these flavonoids, 18 flavone, and 64 flavonol compounds were the main flavonoids. More than half of the 118 flavonoids were glycosides, which are the most abundant form of flavonoids in plants (Ren et al., 2014; Kim et al., 2018; Richardson et al., 2018; Wang et al., 2021). It is worth noting that one isoflavone (daidzein) and one flavone compound (procyanidin) were identified in these fruits for the first time. It has been reported that isoflavones can supple estrogen and have the same human health care functions as other flavonoids, and also function in cancer prevention (Pei et al., 2018; Nakajima and Ohizumi, 2019). Thus far, isoflavones have only been found in some legumes, which are therefore the main source of isoflavones for functional food production (Park et al., 2018). Our identification of isoflavones in the fruit of *A. arguta* Sieb.Zucc. not only provides a new source of isoflavones, but also provides new opportunities for developing food and drug homologous functional health foods using this fruit. We also found that the composition and abundance of flavonoids were significantly different between the two main cultivated varieties at the fruit ripening stage. This not only provides a solid theoretical and practical basis for the comprehensive development of *A. arguta* Sieb.Zucc., but also provides key information for artificial cultivation of *A. arguta* Sieb.Zucc. in Northern China.

Two types of genes are bound up with plant flavonoid biosynthesis (Chen et al., 2017; Nabavi et al., 2020). The first is structural genes encoding enzymes that can catalyze the biosynthesis of different flavonoids; the second is regulatory genes that regulate structural gene transcription (Liu et al., 2016; Nabavi et al., 2020). In this experiment, we used gene functional annotation and KEGG enrichment analysis to determine 41 DEGs encoding enzymes known to be involved in flavonoid biosynthesis: CSUGT134, LOC, AT2, CHS, CHI, C4H, HCT, CCoAOMT, F3H, CFOL, DFR, LAR2, LSAT, and GSCOC. In many plants, naringin, saonol, kaempferol, myricetin, dihydromyricetin, and dihydroquercetin are catalyzed by positively regulating the expression of CHS, CHI, F3H, F3′H, and FLS (Liu et al., 2002; Deavours and Dixon, 2005; Chen et al., 2015; Wang et al., 2017, 2019; Matsui et al., 2018; Zhai et al., 2019). AaF3H, AaLDOX, AaUFGT, AaMYB, AabHLH, and AaHB2 showed the best possibility as candidate genes. The regulatory network of flavonoid biosynthesis was established, indicating that the differentially expressed candidate genes were involved in the accumulation of metabolites (Li et al., 2018). In the KEGG pathway, it is also noted that CHS, CHI, F3H, CFOL, LOC, LSAT, FNSI, DFR, F3′H, FLS, and HIDH may play the role of structure and regulatory genes in the biosynthesis path of flavonoids such as aspen (flavone1), rutin (flavone2), and daidzein (flavone3). In particular, DFR, F3′H, and FLS compete with the substrate dihydrokaempferol DHK. The expression of FLS is relatively high, and its coding enzyme activity is high, which finally promotes the relative content of rutin; the expression of LOC was relatively low, and the relative contents of populin and daidzein were relatively low.

Among these DEGs are key genes involved in flavonoid biosynthesis, namely 3 CSUGT134, 1 LAR2, 1 C4H, 2 CFOL, 1 CHI, 9 LOC, 1 GSCOC, 1 CCoAOMT, 1 DFR, and 2 AT2.

By assessing the transcriptomes, we determined 43,686 genes in *A. arguta* Sieb.Zucc., including 11,650 DEGs. These genes regulate the metabolism, cellular processes, processing of genetic material, environmental inputs, and organizational system of *A. arguta* Sieb.Zucc.

For some structural genes, mRNA expression levels were closely correlated with the accumulation of specific flavonoids, suggesting that this flavonoid biosynthesis gene expression promoted flavonoid accumulation during fruit ripening in *A. arguta* Sieb.Zucc. Here, we found that 1 CHS, 1 F3H, 2 LAR2, 6 CSUGT134, 16 LOC, 2 AT2, 2 C4H, 1 HCT, 1 CCoAOMT, and 1 GSCOC genes were significantly up-regulated, and 1 CHI, 1 LAR2, 2 CFOL, 1 C4H, 1 DFR, 1 LOC, and 1 LSAT genes were significantly down-regulated. We studied the relation between the metabolite and transcriptome data, which demonstrated that the expression of structural genes regulating total flavonoids in Lc varieties was higher than that in Qssg varieties and combining these data was an effective strategy to identify genes bound up with flavonoid biosynthesis.

In conclusion, we here identified flavonoid components present in *A. arguta* Sieb.Zucc. fruits and measured differential gene expression and flavonoid accumulation between the two main cultivars grown in Northern China. Differentially expressed structural genes bound up with flavonoid biosynthesis were identified and validated with qRT-PCR. We also analyzed flavonoid biosynthesis gene expression levels that affect flavonoid accumulation. Through the correlation analysis between transcriptome and metabolite analysis, some flavonoids catalyzed by the expression of CsUGT134, LAR2, C4H, CFOL, CHI, LOC, GSCOC, CCoAOMT, DFR, and AT2 genes were known. These results expand the current understanding of flavonoid composition, accumulation patterns, and biosynthesis mechanisms in *A. arguta* Sieb.Zucc. The data provide valuable new information for future development of homologous functional foods making use of *A. arguta* Sieb.Zucc.

## Data Availability Statement

The datasets presented in this study can be found in online repositories. The names of the repository/repositories and accession number(s) can be found in the article/Supplementary Material.

## Author Contributions

LC designed the project and took part in the project administration and funding acquisition. YbW carried out the experiment and wrote the draft for the first time. JS, HJ, and MI helped to review and edit the manuscript. FY and YD have provided the experimental materials and valuable opinions. LC and YW had overall responsibility for this project, including project ideas, guidance on experimental design, data analysis, manuscript writing, and revision. All authors have read and approved the final manuscript.

## Conflict of Interest

YD was employed by Shandong Xianda Agrochemical Co., Ltd. The remaining authors declare that the research was conducted in the absence of any commercial or financial relationships that could be construed as a potential conflict of interest.

## Publisher’s Note

All claims expressed in this article are solely those of the authors and do not necessarily represent those of their affiliated organizations, or those of the publisher, the editors and the reviewers. Any product that may be evaluated in this article, or claim that may be made by its manufacturer, is not guaranteed or endorsed by the publisher.

## Footnotes

1
http://www.plantcyc.org/

2
http://www.kegg.jp/

3
https://code.google.com/p/corset-project/
