# Supplementary material for: Metabolomic and Transcriptomic Analysis of Flavonoid Biosynthesis in Two Main Cultivars of Actinidia arguta Sieb.Zucc. Grown in Northern China
Source: Front Plant Sci. 2022 Jun 30;13:911203. doi: 10.3389/fpls.2022.911203 (PMC9280664; doi:10.3389/fpls.2022.911203)
Supplement: Supplementary file 1 [file Table_1.DOCX]

| **Table S1 Gene primer information of** Actinidia arguta | | |
| --- | --- | --- |
| Gene Number | Primer Name | Sequence (5'to3') |
| 1 | Internal reference ACTIN-F | CGAACGGGAAATTGTCCGT |
|  | Internal reference ACTIN-R | TTCTCAACTGAGGAGCTGCTCTT |
| 2 | Cluster-10307.10288-F | AGAATGGGGACTTGCTTTAGC |
|  | Cluster-10307.10288-R | CCAAACCAAACCAATTTCACCA |
| 3 | Cluster-10307.30160-F | CGATTGAATCATGCAGACA |
|  | Cluster-10307.30160-R | TCAAGTCCTTGTGATTACCTA |
| 4 | Cluster-10307.4519-F | GTTGGTCCAGAACTTCGA |
|  | Cluster-10307.4519-R | CAGAACAATGGTTGAGTGC |
| 5 | Cluster-10307.5267-F | GAAAGTGGTGGTGGAGAAAGAT |
|  | Cluster-10307.5267-R | ACAAGCCCATCTATCTCTTCCT |
| 6 | Cluster-10307.55417-F | GGGAATAGTTGGGTTGAGATTGG |
|  | Cluster-10307.55417-R | GTGAGTGGAAGTTTTATGGAGACC |
| 7 | Cluster-10307.69071-F | CAGTGTCTCCTTCCTTGAA |
|  | Cluster-10307.69071-R | GCCAGCAACTCAAAAGTAG |
| 8 | Cluster-10307.81571-F | AGTTCAGCCTACACATTGC |
|  | Cluster-10307.81571-R | GGATACAAACAGTACCCCAC |
| 9 | Cluster-10307.9753-F | TCCTTTGTCTTGGAGGTTT |
|  | Cluster-10307.9753-R | GTCGAGCCACTTATCTTCT |
| 10 | Cluster-6365.0-F | ATTTTCTCTCCGCTAACG |
|  | Cluster-6365.0-R | ACCGCATTTAATCTCTTCA |
| 11 | Cluster-10307.4971-F | CCAGTGGTGTATGAAATGAAAC |
|  | Cluster-10307.4971-R | AGAGAAACCATCAAGCCATC |
| 12 | Cluster-10307.82368-F | TCACTCTGAAGAAGGCTCT |
|  | Cluster-10307.82368-R | TCGCAGTAATGGCAACAA |

| Table S2. Statistical list of differential Flavonoids during   Actinidia arguta fruit maturity | | | |
| --- | --- | --- | --- |
| **Qssg vs Lc** | | | |
| Formula | Compounds | Class I | Class II |
| C_15_H_10_O_4_ | Chrysin | Flavonoids | Flavones |
| C_15_H_10_O_5_ | Baicalein | Flavonoids | Flavones |
| C_15_H_12_O_5_ | Pinobanksin* | Flavonoids | Flavanonols |
| C_15_H_12_O_5_ | Naringenin (5,7,4'-Trihydroxyflavanone)* | Flavonoids | Flavanones |
| C_16_H_14_O_5_ | 7-O-Methylnaringenin | Flavonoids | Flavanones |
| C_15_H_12_O_6_ | Aromadendrin (Dihydrokaempferol) | Flavonoids | Flavanonols |
| C_15_H_14_O_6_ | Epicatechin* | Flavonoids | Flavanols |
| C_15_H_14_O_6_ | Catechin* | Flavonoids | Flavanols |
| C_16_H_12_O_6_ | 5,7,2'-Trhiyroxy-8-methoxyflavone* | Flavonoids | Flavones |
| C_16_H_12_O_6_ | Hispidulin (5,7,4'-Trihydroxy-6-methoxyflavone)* | Flavonoids | Flavones |
| C_16_H_12_O_6_ | Diosmetin (5,7,3'-Trihydroxy-4'-methoxyflavone)* | Flavonoids | Flavanones |
| C_15_H_10_O_7_ | Quercetin | Flavonoids | Flavonols |
| C_16_H_14_O_6_ | Dihydrokaempferide | Flavonoids | Flavanonols |
| C_15_H_14_O_7_ | Epigallocatechin | Flavonoids | Flavanols |
| C_17_H_14_O_7_ | Quercetin-3',4'-dimethyl ether | Flavonoids | Flavonols |
| C_20_H_20_O_7_ | Tangeretin (4',5,6,7,8-Pentamethoxyflavone) | Flavonoids | Flavones |
| C_21_H_22_O_8_ | Nobiletin (5,6,7,8,3',4'-Hexamethoxyflavone) | Flavonoids | Flavones |
| C_21_H_20_O_10_ | Apigenin-7-O-glucoside(Cosmosiin) | Flavonoids | Flavones |
| C_21_H_20_O_10_ | Galangin-7-O-glucoside | Flavonoids | Flavones |
| C_20_H_18_O_11_ | Avicularin(Quercetin-3-O-α-L-arabinofuranoside)* | Flavonoids | Flavonols |
| C_20_H_18_O_11_ | Quercetin-3-O-xyloside (Reynoutrin)* | Flavonoids | Flavonols |
| C_20_H_18_O_11_ | Quercetin-3-O-arabinoside (Guaijaverin)* | Flavonoids | Flavonols |
| C_21_H_22_O_10_ | Naringenin-7-O-glucoside (Prunin) | Flavonoids | Flavanones |
| C_21_H_22_O_10_ | Isosalipurposide (Phlorizin Chalcone) | Flavonoids | Chalcones |
| C_21_H_22_O_10_ | Isohemiphloin | Flavonoids | Flavonoid carbonoside |
| C_21_H_24_O_10_ | Phloretin-4'-O-glucoside (Trilobatin) | Flavonoids | Chalcones |
| C_22_H_18_O_10_ | Catechin gallate* | Flavonoids | Flavanols |
| C_22_H_18_O_10_ | Epicatechin gallate* | Flavonoids | Flavanols |
| C_21_H_20_O_11_ | Kaempferol-3-O-glucoside (Astragalin) | Flavonoids | Flavonols |
| C_21_H_20_O_11_ | Kaempferol-7-O-glucoside* | Flavonoids | Flavonols |
| C_21_H_20_O_11_ | Luteolin-4'-O-glucoside* | Flavonoids | Flavones |
| C_21_H_20_O_11_ | Kaempferol-3-O-galactoside (Trifolin)* | Flavonoids | Flavonols |
| C_21_H_20_O_11_ | Luteolin-3'-O-glucoside* | Flavonoids | Flavones |
| C_21_H_22_O_11_ | Aromadendrin-7-O-glucoside | Flavonoids | Flavanonols |
| C_21_H_22_O_11_ | 6-C-Glucosyl-2-Hydroxynaringenin | Flavonoids | Flavanones |
| C_21_H_22_O_11_ | Taxifolin-3-O-rhamnoside (Astilbin) | Flavonoids | Flavanonols |
| C_21_H_22_O_11_ | Dihydrokaempferol-3-O-glucoside | Flavonoids | Flavanonols |
| C_21_H_22_O_11_ | Eriodictyol-3'-O-glucoside | Flavonoids | Flavanones |
| C_24_H_20_O_9_ | Catechin-(7,8-bc)-4α-(3,4-dihydroxyphenyl)-dihydro-2-(3H)-one* | Flavonoids | Flavanols |
| C_24_H_20_O_9_ | Catechin-(7,8-bc)-4β-(3,4-dihydroxyphenyl)-dihydro-2-(3H)-one* | Flavonoids | Flavanols |
| C_21_H_24_O_11_ | Epicatechin glucoside | Flavonoids | Flavanols |
| C_22_H_18_O_11_ | Epigallocatechin-3-gallate* | Flavonoids | Flavanols |
| C_22_H_18_O_11_ | Gallocatechin 3-O-gallate* | Flavonoids | Flavanols |
| C_22_H_22_O_11_ | Diosmetin-7-O-glucoside* | Flavonoids | Flavanones |
| C_22_H_22_O_11_ | Diosmetin-7-O-galactoside* | Flavonoids | Flavanones |
| C_22_H_22_O_11_ | Hispidulin-7-O-Glucoside | Flavonoids | Flavones |
| C_21_H_20_O_12_ | 6-Hydroxykaempferol-7-O-glucoside | Flavonoids | Flavonols |
| C_21_H_20_O_12_ | Isohyperoside | Flavonoids | Flavonols |
| C_21_H_20_O_12_ | 6-Hydroxyluteolin 5-glucoside | Flavonoids | Flavones |
| C_21_H_20_O_12_ | Quercetin-3-O-galactoside (Hyperin)* | Flavonoids | Flavonols |
| C_21_H_20_O_12_ | Quercetin-3-O-glucoside (Isoquercitrin)* | Flavonoids | Flavonols |
| C_21_H_20_O_12_ | Quercetin-4'-O-glucoside (Spiraeoside)* | Flavonoids | Flavonols |
| C_21_H_20_O_12_ | Quercetin-7-O-glucoside* | Flavonoids | Flavonols |
| C_22_H_24_O_11_ | Hesperetin-7-O-glucoside | Flavonoids | Flavanones |
| C_22_H_24_O_11_ | Hesperetin-5-O-glucoside | Flavonoids | Flavanones |
| C_23_H_16_O_11_ | Isorhamnetin-3-O-gallate | Flavonoids | Flavonols |
| C_22_H_22_O_12_ | Isorhamnetin-3-O-Glucoside* | Flavonoids | Flavonols |
| C_22_H_22_O_12_ | Rhamnetin-3-O-Glucoside* | Flavonoids | Flavonols |
| C_22_H_22_O_12_ | 6-Methoxykaempferol-3-O-glucoside | Flavonoids | Flavonols |
| C_22_H_22_O_12_ | Isorhamnetin-7-O-glucoside (Brassicin)* | Flavonoids | Flavonols |
| C_22_H_22_O_13_ | Mearnsetin-3-O-glucoside | Flavonoids | Flavones |
| C_23_H_22_O_13_ | Quercetin-3-O-(6''-acetyl)galactoside | Flavonoids | Flavonols |
| C_24_H_22_O_14_ | Kaempferol-3-O-(6''-malonyl)glucoside* | Flavonoids | Flavonols |
| C_24_H_22_O_14_ | Kaempferol-3-O-(6''-malonyl)galactoside* | Flavonoids | Flavonols |
| C_24_H_22_O_15_ | Quercetin-3-O-(6''-malonyl)galactoside | Flavonoids | Flavonols |
| C_24_H_22_O_15_ | Quercetin-7-O-(6''-malonyl)glucoside | Flavonoids | Flavonols |
| C_25_H_26_O_14_ | Hesperetin-7-O-(6''-malonyl)glucoside | Flavonoids | Flavanones |
| C_30_H_26_O_11_ | Epicatechin-epiafzelechin | Flavonoids | Flavanols |
| C_26_H_26_O_15_ | Apigenin-6-C-(2''-glucuronyl)xyloside | Flavonoids | Flavonoid carbonoside |
| C_27_H_30_O_14_ | Kaempferol-3,7-O-dirhamnoside (Kaempferitrin) | Flavonoids | Flavonols |
| C_26_H_28_O_15_ | Luteolin-7-O-glucoside-5-O-arabinoside | Flavonoids | Flavones |
| C_30_H_26_O_13_ | Kaempferol-3-O-(6''-p-Coumaroyl)galactoside | Flavonoids | Flavonols |
| C_30_H_26_O_13_ | Kaempferol-3-O-(6''-p-Coumaroyl)glucoside (Tiliroside) | Flavonoids | Flavonols |
| C_30_H_26_O_13_ | Luteolin-7-O-(6''-caffeoyl)rhamnoside | Flavonoids | Flavones |
| C_27_H_30_O_15_ | Luteolin-7-O-neohesperidoside (Lonicerin) | Flavonoids | Flavones |
| C_27_H_30_O_15_ | Kaempferol-3-O-robinobioside(Biorobin) | Flavonoids | Flavonols |
| C_27_H_30_O_15_ | Kaempferol-3-O-neohesperidoside* | Flavonoids | Flavonols |
| C_27_H_30_O_15_ | Kaempferol-3-O-rutinoside(Nicotiflorin)* | Flavonoids | Flavonols |
| C_27_H_30_O_15_ | Luteolin-7-O-rutinoside | Flavonoids | Flavones |
| C_27_H_30_O_15_ | Quercetin-3,7-Di-O-rhamnoside | Flavonoids | Flavonols |
| C_27_H_30_O_15_ | Kaempferol-3-O-glucorhamnoside | Flavonoids | Flavonols |
| C_28_H_34_O_14_ | Poncirin (Isosakuranetin-7-O-neohesperidoside) | Flavonoids | Flavanones |
| C_26_H_28_O_16_ | Quercetin-3-O-(6''-O-arabinosyl)glucoside | Flavonoids | Flavonols |
| C_26_H_28_O_16_ | Quercetin-3-O-sambubioside* | Flavonoids | Flavonols |
| C_26_H_28_O_16_ | Quercetin-3-O-apiosyl(1→2)galactoside* | Flavonoids | Flavonols |
| C_30_H_26_O_14_ | Quercetin-3-O-(6''-p-Coumaroyl)glucoside* | Flavonoids | Flavonols |
| C_30_H_26_O_14_ | Quercetin-3-O-(6''-p-Coumaroyl)galactoside* | Flavonoids | Flavonols |
| C_27_H_30_O_16_ | Quercetin-3-O-(2''-O-rhamnosyl)galactoside | Flavonoids | Flavonols |
| C_27_H_30_O_16_ | Quercetin-7-O-rutinoside* | Flavonoids | Flavonols |
| C_27_H_30_O_16_ | Orientin-7-O-glucoside | Flavonoids | Flavonoid carbonoside |
| C_27_H_30_O_16_ | Quercetin-3-O-rutinoside (Rutin)* | Flavonoids | Flavonols |
| C_27_H_30_O_16_ | Quercetin-3-O-(4''-O-glucosyl)rhamnoside* | Flavonoids | Flavonols |
| C_27_H_30_O_16_ | Quercetin-3-O-glucoside-7-O-rhamnoside* | Flavonoids | Flavonols |
| C_27_H_30_O_16_ | Isorhamnetin-3-O-(2''-O-xylosyl)glucoside | Flavonoids | Flavonols |
| C_27_H_30_O_16_ | Quercetin-3-O-robinobioside* | Flavonoids | Flavonols |
| C_28_H_32_O_16_ | Rhamnetin-3-O-Rutinoside* | Flavonoids | Flavonols |
| C_28_H_32_O_16_ | Isorhamnetin-3-O-rutinoside (Narcissin)* | Flavonoids | Flavonols |
| C_28_H_32_O_16_ | Isorhamnetin-3-O-neohespeidoside* | Flavonoids | Flavonols |
| C_27_H_30_O_17_ | Quercetin-3-O-sophoroside (Baimaside) | Flavonoids | Flavonols |
| C_27_H_30_O_17_ | Meratin | Flavonoids | Flavones |
| C_27_H_30_O_17_ | Quercetin-5,4ʹ-di-O-glucoside | Flavonoids | Flavonols |
| C_27_H_30_O_17_ | 6-Hydroxykaempferol-3,6-O-Diglucoside | Flavonoids | Flavonols |
| C_27_H_30_O_17_ | Quercetin-3-O-(2''-O-galactosyl)glucoside | Flavonoids | Flavonols |
| C_29_H_30_O_19_ | Quercetin-3-O-(2''-malonyl)glucoside-7-O-arabinoside | Flavonoids | Flavonols |
| C_30_H_32_O_20_ | Quercetin-7-O-(2''-malonyl)glucosyl-5-O-glucoside | Flavonoids | Flavonols |
| C_32_H_38_O_19_ | Kaempferol-3-O-(2-O-Xylosyl-6-O-Rhamnosyl)Glucoside | Flavonoids | Flavonols |
| C_32_H_38_O_20_ | Quercetin-3-O-(2''-O-Xylosyl)rutinoside | Flavonoids | Flavonols |
| C_32_H_38_O_20_ | Quercetin-3-O-(2''-O-arabinosyl)rutinoside | Flavonoids | Flavonols |
| C_33_H_40_O_20_ | Quercetin-3-O-rutinoside-7-O-rhamnoside | Flavonoids | Flavonols |
| C_33_H_40_O_20_ | Kaempferol-3-O-(6''-Rhamnosyl-2''-Glucosyl)Glucoside (Camelliaside A) | Flavonoids | Flavonols |
| C_33_H_40_O_20_ | Quercetin-3-O-(2''-O-Rhamnosyl)rutinoside | Flavonoids | Flavonols |
| C_34_H_42_O_20_ | Rhamnetin-3-O-Rutinoside-5-O-rhamnoside* | Flavonoids | Flavonols |
| C_34_H_42_O_20_ | Isorhamnetin-3-O-rutinoside-7-O-rhamnoside* | Flavonoids | Flavonols |
| C_33_H_40_O_21_ | 6-Hydroxykaempferol 3-Rutinoside -6-glucoside | Flavonoids | Flavonols |
| C_33_H_40_O_21_ | Kaempferol-3-O-sophorotrioside | Flavonoids | Flavonols |
| C_33_H_40_O_22_ | 6-Hydroxykaempferol-3,7,6-O-triglycoside | Flavonoids | Flavonols |
| C_45_H_38_O_18_ | Catechin-catechin-catechin | Flavonoids | Flavanols |
| C_15_H_10_O_4_ | Daidzein | Flavonoids | Isoflavones |

| **Table S3. Significantly changed metabolites in Qssg compared with Lc** | | | | | | | | | | |  |
| --- | --- | --- | --- | --- | --- | --- | --- | --- | --- | --- | --- |
| Class | Compounds | Qssg-1 | Qssg-2 | Qssg-3 | Lc-1 | Lc-2 | Lc-3 | Log2(FC) | p-value | VIP | Type |
| Flavonols | Quercetin-3,7-Di-O-rhamnoside | 9 | 9 | 9 | 9820 | 4170 | 4200 | -9.39597042 | 0.08426651 | 1.706248462 | down |
|  | Quercetin-3-O-(6''-p-Coumaroyl)glucoside* | 1360 | 6790 | 604 | 14300 | 4240 | 8300 | -1.616370382 | 0.171675747 | 1.152937924 | down |
|  | Quercetin-3-O-(2''-O-Xylosyl)rutinoside | 9 | 705 | 711 | 24600 | 6290 | 15400 | -5.021666738 | 0.105229619 | 1.415162334 | down |
|  | 6-Hydroxykaempferol 3-Rutinoside -6-glucoside | 26000 | 8000 | 17400 | 9720 | 8150 | 6730 | 1.063110044 | 0.225536503 | 1.104665409 | up |
|  | 6-Hydroxykaempferol-3,7,6-O-triglycoside | 22100 | 8530 | 13200 | 2950 | 8850 | 4720 | 1.407704992 | 0.135711288 | 1.31905374 | up |
|  | Quercetin-5,4ʹ-di-O-glucoside | 4710 | 13000 | 2940 | 30000 | 11100 | 25100 | -1.680689435 | 0.097325987 | 1.300996143 | down |
|  | Isorhamnetin-3-O-(2''-O-xylosyl)glucoside | 5270 | 5940 | 8310 | 11200 | 15000 | 61600 | -2.169267887 | 0.295139992 | 1.301674012 | down |
|  | Quercetin-3-O-(6''-p-Coumaroyl)galactoside* | 15300 | 41700 | 11400 | 72800 | 20000 | 56200 | -1.1232441 | 0.229412016 | 1.000419799 | down |
|  | Quercetin-3-O-(2''-malonyl)glucoside-7-O-arabinoside | 1390 | 2160 | 3630 | 51200 | 20300 | 45500 | -4.026380876 | 0.060495539 | 1.659437274 | down |
|  | Isorhamnetin-3-O-gallate | 50900 | 88900 | 67000 | 23500 | 33100 | 17800 | 1.474861659 | 0.042048354 | 1.573509158 | up |
|  | Rhamnetin-3-O-Glucoside* | 287000 | 243000 | 138000 | 199000 | 80900 | 35500 | 1.082665443 | 0.149220829 | 1.133071199 | up |
|  | Isorhamnetin-3-O-Glucoside* | 288000 | 199000 | 146000 | 165000 | 85600 | 35000 | 1.14820952 | 0.108532542 | 1.198555785 | up |
|  | Quercetin-3-O-(2''-O-rhamnosyl)galactoside | 9 | 9 | 9 | 402000 | 133000 | 152000 | -14.63506307 | 0.118362056 | 1.708704266 | down |
|  | Quercetin-3-O-(6''-O-arabinosyl)glucoside | 4340 | 1240 | 2290 | 557000 | 159000 | 432000 | -7.188543291 | 0.083759804 | 1.685824881 | down |
|  | 6-Hydroxykaempferol-3,6-O-Diglucoside | 435000 | 136000 | 320000 | 176000 | 165000 | 94300 | 1.033415412 | 0.217645135 | 1.053551695 | up |
|  | Kaempferol-3-O-(2-O-Xylosyl-6-O-Rhamnosyl)Glucoside | 7560 | 9 | 1510 | 250000 | 226000 | 110000 | -6.012223453 | 0.046627045 | 1.434513778 | down |
|  | Kaempferol-3-O-glucorhamnoside | 899000 | 292000 | 1400000 | 309000 | 260000 | 108000 | 1.936281277 | 0.180937239 | 1.242682428 | up |
|  | Kaempferol-3-O-neohesperidoside* | 1300000 | 218000 | 1670000 | 364000 | 295000 | 72100 | 2.124510972 | 0.197060569 | 1.078346684 | up |
|  | Kaempferol-3-O-rutinoside(Nicotiflorin)* | 1130000 | 252000 | 1540000 | 328000 | 303000 | 108000 | 1.983309909 | 0.192209023 | 1.143402024 | up |
|  | Quercetin-3-O-(2''-O-arabinosyl)rutinoside | 16800 | 1410 | 4180 | 1940000 | 537000 | 982000 | -7.271356675 | 0.109514497 | 1.651471501 | down |
|  | Quercetin-3-O-apiosyl(1→2)galactoside* | 26500 | 7560 | 1800 | 1810000 | 601000 | 1350000 | -6.712597008 | 0.071862714 | 1.621569577 | down |
|  | Quercetin-3-O-sambubioside* | 9 | 9 | 9 | 2250000 | 603000 | 1500000 | -17.29869109 | 0.09289618 | 1.710419951 | down |
| Flavones | Apigenin-7-O-glucoside(Cosmosiin) | 12100 | 13800 | 4740 | 37400 | 10100 | 33100 | -1.395363541 | 0.180081787 | 1.119550669 | down |
|  | Luteolin-7-O-glucoside-5-O-arabinoside | 670 | 8760 | 5860 | 11700 | 11000 | 16000 | -1.33974516 | 0.060129357 | 1.099381604 | down |
|  | Galangin-7-O-glucoside | 3070 | 7960 | 11400 | 37400 | 14700 | 22100 | -1.725989566 | 0.110027535 | 1.383867703 | down |
|  | Luteolin-7-O-neohesperidoside (Lonicerin) | 1170000 | 207000 | 1360000 | 366000 | 250000 | 84900 | 1.965314905 | 0.192837578 | 1.073894808 | up |
| Flavanones | Hesperetin-7-O-(6''-malonyl)glucoside | 18000 | 36600 | 26100 | 8180 | 3750 | 9780 | 1.894208948 | 0.055439312 | 1.499534507 | up |
|  | Eriodictyol-3'-O-glucoside | 15200 | 13800 | 10400 | 38500 | 31500 | 22600 | -1.232816564 | 0.050712044 | 1.543080329 | down |
|  | Naringenin-7-O-glucoside (Prunin) | 35200 | 44300 | 22100 | 96600 | 53200 | 128000 | -1.451146197 | 0.103463377 | 1.423392138 | down |
|  | 6-C-Glucosyl-2-Hydroxynaringenin | 969000 | 875000 | 1030000 | 5630000 | 5610000 | 5970000 | -2.58211513 | 0.000126161 | 1.711918514 | down |
| Flavanols | Catechin-(7,8-bc)-4α-(3,4-dihydroxyphenyl)-dihydro-2-(3H)-one* | 2200 | 20300 | 6510 | 112000 | 31100 | 20300 | -2.493785783 | 0.259550789 | 1.260475053 | down |
|  | Catechin-(7,8-bc)-4β-(3,4-dihydroxyphenyl)-dihydro-2-(3H)-one* | 2900 | 30600 | 5480 | 73800 | 16800 | 19100 | -1.49275753 | 0.339594582 | 1.020961752 | down |
|  | Epicatechin-epiafzelechin | 14100 | 12200 | 12500 | 64900 | 25800 | 31500 | -1.655115728 | 0.14992319 | 1.525419062 | down |
|  | Epicatechin glucoside | 416000 | 161000 | 119000 | 111000 | 81100 | 65500 | 1.433954713 | 0.254561222 | 1.253868697 | up |
| Chalcones | Isosalipurposide (Phlorizin Chalcone) | 40700 | 104000 | 43100 | 237000 | 116000 | 179000 | -1.502229183 | 0.060541894 | 1.412663607 | down |
|  | Phloretin-4'-O-glucoside (Trilobatin) | 150000 | 135000 | 134000 | 590000 | 310000 | 332000 | -1.555980107 | 0.094176416 | 1.597899484 | down |
| Flavonoid carbonoside | Isohemiphloin | 6510 | 12600 | 2910 | 26100 | 15500 | 22600 | -1.543758828 | 0.029250496 | 1.351641451 | down |
|  | Apigenin-6-C-(2''-glucuronyl)xyloside | 10800 | 3490 | 5100 | 24100 | 14500 | 33400 | -1.892684103 | 0.068790654 | 1.490763598 | down |
| Flavanonol | Taxifolin-3-O-rhamnoside (Astilbin) | 1400 | 16700 | 4300 | 29700 | 37600 | 21700 | -1.990306604 | 0.027748769 | 1.307034628 | down |

| **Table S4. Summary Statistics of Results during *Actinidia arguta* fruit maturity** | | | | | | | |
| --- | --- | --- | --- | --- | --- | --- | --- |
| Sample | Raw Reads | Clean Reads | Clean Base(G) | Error Rate(%) | Q20(%) | Q30(%) | GC Content(%) |
| Lc2-1 | 43976908 | 41844326 | 6.28 | 0.03 | 97.66 | 93.28 | 47.32 |
| Lc2-2 | 47501280 | 45998202 | 6.9 | 0.03 | 97.11 | 91.94 | 47.21 |
| Lc2-3 | 48528214 | 46501380 | 6.98 | 0.03 | 97.11 | 92 | 47.27 |
| Qssg-1 | 51274090 | 48936740 | 7.34 | 0.03 | 97.67 | 93.32 | 47.98 |
| Qssg-2 | 46897420 | 44743014 | 6.71 | 0.03 | 97.16 | 92.15 | 47.62 |
| Qssg-3 | 43580384 | 41708692 | 6.26 | 0.03 | 96.87 | 91.49 | 47.4 |

| **Table S5. List of GO enrichment of DEGs during  *Actinidia arguta* fruit maturity** | | | | |
| --- | --- | --- | --- | --- |
| Ontology | ID | Description | Count | GeneRatio |
| Cellular component | GO:0016020 | membrane | 3590 | 3590/11650 |
| Cellular component | GO:0005576 | extracellular region | 380 | 380/11650 |
| Cellular component | GO:0005623 | cell | 6598 | 6598/11650 |
| Cellular component | GO:0009295 | nucleoid | 10 | 10/11650 |
| Cellular component | GO:0030054 | cell junction | 387 | 387/11650 |
| Cellular component | GO:0031974 | membrane-enclosed lumen | 403 | 403/11650 |
| Cellular component | GO:0032991 | protein-containing complex | 883 | 883/11650 |
| Cellular component | GO:0043226 | organelle | 4909 | 4909/11650 |
| Cellular component | GO:0044215 | other organism | 1 | 1/11650 |
| Cellular component | GO:0044217 | other organism part | 1 | 1/11650 |
| Cellular component | GO:0044421 | extracellular region part | 55 | 55/11650 |
| Cellular component | GO:0044422 | organelle part | 2204 | 2204/11650 |
| Cellular component | GO:0044425 | membrane part | 2674 | 2674/11650 |
| Cellular component | GO:0044464 | cell part | 6594 | 6594/11650 |
| Cellular component | GO:0055044 | symplast | 387 | 387/11650 |
| Cellular component | GO:0099080 | supramolecular complex | 115 | 115/11650 |
| Biological process | GO:0000003 | reproduction | 726 | 726/11650 |
| Biological process | GO:0008152 | metabolic process | 4700 | 4700/11650 |
| Biological process | GO:0001906 | cell killing | 10 | 10/11650 |
| Biological process | GO:0002376 | immune system process | 284 | 284/11650 |
| Biological process | GO:0006791 | sulfur utilization | 1 | 1/11650 |
| Biological process | GO:0040007 | growth | 309 | 309/11650 |
| Biological process | GO:0008283 | cell proliferation | 31 | 31/11650 |
| Biological process | GO:0009987 | cellular process | 5343 | 5343/11650 |
| Biological process | GO:0015976 | carbon utilization | 4 | 4/11650 |
| Biological process | GO:0019740 | nitrogen utilization | 2 | 2/11650 |
| Biological process | GO:0022414 | reproductive process | 722 | 722/11650 |
| Biological process | GO:0022610 | biological adhesion | 17 | 17/11650 |
| Biological process | GO:0023052 | signaling | 1080 | 1080/11650 |
| Biological process | GO:0032501 | multicellular organismal process | 1231 | 1231/11650 |
| Biological process | GO:0032502 | developmental process | 1347 | 1347/11650 |
| Biological process | GO:0040011 | locomotion | 18 | 18/11650 |
| Biological process | GO:0043473 | pigmentation | 1 | 1/11650 |
| Biological process | GO:0048511 | rhythmic process | 114 | 114/11650 |
| Biological process | GO:0048518 | positive regulation of biological process | 509 | 509/11650 |
| Biological process | GO:0048519 | negative regulation of biological process | 590 | 590/11650 |
| Biological process | GO:0050789 | regulation of biological process | 2442 | 2442/11650 |
| Biological process | GO:0050896 | response to stimulus | 2939 | 2939/11650 |
| Biological process | GO:0051179 | localization | 989 | 989/11650 |
| Biological process | GO:0051704 | multi-organism process | 728 | 728/11650 |
| Biological process | GO:0065007 | biological regulation | 2704 | 2704/11650 |
| Biological process | GO:0071840 | cellular component organization or biogenesis | 1077 | 1077/11650 |
| Biological process | GO:0098754 | detoxification | 16 | 16/11650 |
| Molecular function | GO:0140110 | transcription regulator activity | 734 | 734/11650 |
| Molecular function | GO:0003824 | catalytic activity | 4376 | 4376/11650 |
| Molecular function | GO:0045735 | nutrient reservoir activity | 34 | 34/11650 |
| Molecular function | GO:0005198 | structural molecule activity | 150 | 150/11650 |
| Molecular function | GO:0005215 | transporter activity | 518 | 518/11650 |
| Molecular function | GO:0005488 | binding | 4940 | 4940/11650 |
| Molecular function | GO:0031386 | protein tag | 1 | 1/11650 |
| Molecular function | GO:0016209 | antioxidant activity | 63 | 63/11650 |
| Molecular function | GO:0098772 | molecular function regulator | 171 | 171/11650 |
| Molecular function | GO:0045182 | translation regulator activity | 1 | 1/11650 |
| Molecular function | GO:0060089 | molecular transducer activity | 164 | 164/11650 |
| Molecular function | GO:0140104 | molecular carrier activity | 10 | 10/11650 |

| **Table S6. List of KEGG pathway enrichment of DEGs during**  **Actinidia arguta fruit maturity** | | |
| --- | --- | --- |
| **Qssg vs Lc** | | |
| **Total_gene** | | 4024 |
| **Pathway** | **ko_id** | **DEG No.** |
| Metabolic pathways | ko01100 | 1766 |
| Biosynthesis of secondary metabolites | ko01110 | 1007 |
| MAPK signaling pathway - plant | ko04016 | 284 |
| Starch and sucrose metabolism | ko00500 | 207 |
| Plant hormone signal transduction | ko04075 | 356 |
| Phenylpropanoid biosynthesis | ko00940 | 103 |
| Plant-pathogen interaction | ko04626 | 402 |
| Sesquiterpenoid and triterpenoid biosynthesis | ko00909 | 31 |
| Cyanoamino acid metabolism | ko00460 | 42 |
| Cysteine and methionine metabolism | ko00270 | 115 |
| Glycosaminoglycan degradation | ko00531 | 38 |
| Glycosphingolipid biosynthesis - ganglio series | ko00604 | 28 |
| alpha-Linolenic acid metabolism | ko00592 | 38 |
| Terpenoid backbone biosynthesis | ko00900 | 50 |
| Flavonoid biosynthesis | ko00941 | 35 |
| Cutin, suberine and wax biosynthesis | ko00073 | 21 |
| Synthesis and degradation of ketone bodies | ko00072 | 13 |
| Diterpenoid biosynthesis | ko00904 | 25 |
| Butanoate metabolism | ko00650 | 24 |
| Monoterpenoid biosynthesis | ko00902 | 15 |
| Pentose and glucuronate interconversions | ko00040 | 82 |
| Stilbenoid, diarylheptanoid and gingerol biosynthesis | ko00945 | 19 |
| Alanine, aspartate and glutamate metabolism | ko00250 | 44 |
| Other glycan degradation | ko00511 | 49 |
| Valine, leucine and isoleucine degradation | ko00280 | 58 |
| Glutathione metabolism | ko00480 | 57 |
| Phenylalanine, tyrosine and tryptophan biosynthesis | ko00400 | 48 |
| Linoleic acid metabolism | ko00591 | 19 |
| Photosynthesis - antenna proteins | ko00196 | 18 |
| Glucosinolate biosynthesis | ko00966 | 7 |
| Glycerolipid metabolism | ko00561 | 70 |
| Selenocompound metabolism | ko00450 | 18 |
| Arachidonic acid metabolism | ko00590 | 24 |
| Isoquinoline alkaloid biosynthesis | ko00950 | 27 |
| Glycerophospholipid metabolism | ko00564 | 89 |
| Glyoxylate and dicarboxylate metabolism | ko00630 | 74 |
| Tropane, piperidine and pyridine alkaloid biosynthesis | ko00960 | 26 |
| Isoflavonoid biosynthesis | ko00943 | 18 |
| Biosynthesis of unsaturated fatty acids | ko01040 | 16 |
| Galactose metabolism | ko00052 | 76 |
| Ether lipid metabolism | ko00565 | 33 |
| Nitrogen metabolism | ko00910 | 18 |
| Carotenoid biosynthesis | ko00906 | 27 |
| Biosynthesis of amino acids | ko01230 | 185 |
| Phosphatidylinositol signaling system | ko04070 | 69 |
| ABC transporters | ko02010 | 60 |
| Glycosphingolipid biosynthesis - lacto and neolacto series | ko00601 | 3 |
| Fructose and mannose metabolism | ko00051 | 66 |
| Benzoxazinoid biosynthesis | ko00402 | 10 |
| Zeatin biosynthesis | ko00908 | 23 |
| Sphingolipid metabolism | ko00600 | 42 |
| Brassinosteroid biosynthesis | ko00905 | 11 |
| Indole alkaloid biosynthesis | ko00901 | 7 |
| Pantothenate and CoA biosynthesis | ko00770 | 27 |
| Amino sugar and nucleotide sugar metabolism | ko00520 | 124 |
| Phenylalanine metabolism | ko00360 | 35 |
| Steroid biosynthesis | ko00100 | 23 |
| Phosphonate and phosphinate metabolism | ko00440 | 5 |
| beta-Alanine metabolism | ko00410 | 37 |
| Ubiquinone and other terpenoid-quinone biosynthesis | ko00130 | 33 |
| Sulfur metabolism | ko00920 | 21 |
| Flavone and flavonol biosynthesis | ko00944 | 6 |
| Inositol phosphate metabolism | ko00562 | 56 |
| Glycine, serine and threonine metabolism | ko00260 | 54 |
| Tyrosine metabolism | ko00350 | 35 |
| Aflatoxin biosynthesis | ko00254 | 3 |
| One carbon pool by folate | ko00670 | 18 |
| Taurine and hypotaurine metabolism | ko00430 | 7 |
| Nicotinate and nicotinamide metabolism | ko00760 | 21 |
| Arginine biosynthesis | ko00220 | 18 |
| Protein processing in endoplasmic reticulum | ko04141 | 225 |
| Carbon fixation in photosynthetic organisms | ko00710 | 68 |
| Peroxisome | ko04146 | 67 |
| Caffeine metabolism | ko00232 | 2 |
| Arginine and proline metabolism | ko00330 | 41 |
| Porphyrin and chlorophyll metabolism | ko00860 | 37 |
| Glycosphingolipid biosynthesis - globo and isoglobo series | ko00603 | 4 |
| Anthocyanin biosynthesis | ko00942 | 4 |
| Mannose type O-glycan biosynthesis | ko00515 | 1 |
| Fatty acid metabolism | ko01212 | 43 |
| Ascorbate and aldarate metabolism | ko00053 | 29 |
| Monobactam biosynthesis | ko00261 | 9 |
| Fatty acid degradation | ko00071 | 26 |
| Biotin metabolism | ko00780 | 13 |
| Valine, leucine and isoleucine biosynthesis | ko00290 | 8 |
| Thiamine metabolism | ko00730 | 14 |
| Riboflavin metabolism | ko00740 | 12 |
| Pyrimidine metabolism | ko00240 | 46 |
| Other types of O-glycan biosynthesis | ko00514 | 10 |
| N-Glycan biosynthesis | ko00510 | 40 |
| Phagosome | ko04145 | 68 |
| Lysine biosynthesis | ko00300 | 8 |
| Fatty acid elongation | ko00062 | 10 |
| Basal transcription factors | ko03022 | 26 |
| Base excision repair | ko03410 | 22 |
| Pentose phosphate pathway | ko00030 | 40 |
| Tryptophan metabolism | ko00380 | 32 |
| Citrate cycle (TCA cycle) | ko00020 | 47 |
| Carbon metabolism | ko01200 | 190 |
| Fatty acid biosynthesis | ko00061 | 24 |
| Ubiquitin mediated proteolysis | ko04120 | 136 |
| 2-Oxocarboxylic acid metabolism | ko01210 | 28 |
| Propanoate metabolism | ko00640 | 22 |
| Purine metabolism | ko00230 | 69 |
| Endocytosis | ko04144 | 160 |
| Protein export | ko03060 | 39 |
| Sulfur relay system | ko04122 | 3 |
| Betalain biosynthesis | ko00965 | 2 |
| Vitamin B6 metabolism | ko00750 | 4 |
| Folate biosynthesis | ko00790 | 11 |
| Limonene and pinene degradation | ko00903 | 2 |
| Non-homologous end-joining | ko03450 | 1 |
| C5-Branched dibasic acid metabolism | ko00660 | 1 |
| Glycolysis / Gluconeogenesis | ko00010 | 83 |
| Photosynthesis | ko00195 | 21 |
| Pyruvate metabolism | ko00620 | 67 |
| Circadian rhythm - plant | ko04712 | 44 |
| Various types of N-glycan biosynthesis | ko00513 | 22 |
| Lipoic acid metabolism | ko00785 | 1 |
| Aminoacyl-tRNA biosynthesis | ko00970 | 35 |
| RNA polymerase | ko03020 | 14 |
| Histidine metabolism | ko00340 | 4 |
| Glycosylphosphatidylinositol (GPI)-anchor biosynthesis | ko00563 | 8 |
| DNA replication | ko03030 | 12 |
| Ribosome biogenesis in eukaryotes | ko03008 | 58 |
| Nucleotide excision repair | ko03420 | 23 |
| Oxidative phosphorylation | ko00190 | 64 |
| Lysine degradation | ko00310 | 17 |
| Mismatch repair | ko03430 | 9 |
| SNARE interactions in vesicular transport | ko04130 | 17 |
| RNA degradation | ko03018 | 62 |
| Homologous recombination | ko03440 | 22 |
| Proteasome | ko03050 | 32 |
| Spliceosome | ko03040 | 151 |
| Autophagy - other | ko04136 | 14 |
| mRNA surveillance pathway | ko03015 | 68 |
| Ribosome | ko03010 | 107 |
| RNA transport | ko03013 | 83 |

| **Table S7. List of significantly enriched KEGG pathways of DEGs during**  Actinidia arguta fruit maturity | | |
| --- | --- | --- |
| **Qssg vs Lc** | | |
| **Kegg_pathway** | **ko_id** | **P-value** |
| Metabolic pathways | ko01100 | 0.000000000 |
| Biosynthesis of secondary metabolites | ko01110 | 0.000000000 |
| MAPK signaling pathway - plant | ko04016 | 0.000000000 |
| [Starch and sucrose metabolism](D:\\%E7%8E%8B%E7%A6%B9%E5%8D%9A\\%E7%A6%B9%E5%8D%9A%E5%8D%9A%E5%A3%AB%E5%8F%91%E8%A1%A8%E6%96%87%E7%AB%A0\\%E5%8F%91%E8%A1%A8SCI%E6%96%87%E7%AB%A0\\2021\\%E6%80%BB%E5%8C%96%E5%90%88%E7%89%A9%E8%BD%AC%E5%BD%95%E4%BB%A3%E8%B0%A2%E5%8F%8A%E8%81%94%E5%90%88%E5%88%86%E6%9E%90\\MWXS-21-2190D_%E8%BE%BD%E5%AE%81%E7%9C%81%E7%A7%91%E6%8A%80%E5%A4%A7%E5%AD%A66%E4%B8%AA%E8%BD%AF%E6%9E%A3%E7%8C%95%E7%8C%B4%E6%A1%83%E8%BD%AC%E5%BD%95%E7%BB%84%E6%B5%8B%E5%BA%8F%E6%8A%80%E6%9C%AF%E6%9C%8D%E5%8A%A1%E6%8A%A5%E5%91%8A_20211126\\MWXS-21-2190D\\06.Differential_analysis\\Lc2_vs_Qssg\\pathway_enrichment\\maps\\ko00500.html) | ko00500 | 0.000000001 |
| [Plant hormone signal transduction](D:\\%E7%8E%8B%E7%A6%B9%E5%8D%9A\\%E7%A6%B9%E5%8D%9A%E5%8D%9A%E5%A3%AB%E5%8F%91%E8%A1%A8%E6%96%87%E7%AB%A0\\%E5%8F%91%E8%A1%A8SCI%E6%96%87%E7%AB%A0\\2021\\%E6%80%BB%E5%8C%96%E5%90%88%E7%89%A9%E8%BD%AC%E5%BD%95%E4%BB%A3%E8%B0%A2%E5%8F%8A%E8%81%94%E5%90%88%E5%88%86%E6%9E%90\\MWXS-21-2190D_%E8%BE%BD%E5%AE%81%E7%9C%81%E7%A7%91%E6%8A%80%E5%A4%A7%E5%AD%A66%E4%B8%AA%E8%BD%AF%E6%9E%A3%E7%8C%95%E7%8C%B4%E6%A1%83%E8%BD%AC%E5%BD%95%E7%BB%84%E6%B5%8B%E5%BA%8F%E6%8A%80%E6%9C%AF%E6%9C%8D%E5%8A%A1%E6%8A%A5%E5%91%8A_20211126\\MWXS-21-2190D\\06.Differential_analysis\\Lc2_vs_Qssg\\pathway_enrichment\\maps\\ko04075.html) | ko04075 | 0.000000001 |
| [Phenylpropanoid biosynthesis](D:\\%E7%8E%8B%E7%A6%B9%E5%8D%9A\\%E7%A6%B9%E5%8D%9A%E5%8D%9A%E5%A3%AB%E5%8F%91%E8%A1%A8%E6%96%87%E7%AB%A0\\%E5%8F%91%E8%A1%A8SCI%E6%96%87%E7%AB%A0\\2021\\%E6%80%BB%E5%8C%96%E5%90%88%E7%89%A9%E8%BD%AC%E5%BD%95%E4%BB%A3%E8%B0%A2%E5%8F%8A%E8%81%94%E5%90%88%E5%88%86%E6%9E%90\\MWXS-21-2190D_%E8%BE%BD%E5%AE%81%E7%9C%81%E7%A7%91%E6%8A%80%E5%A4%A7%E5%AD%A66%E4%B8%AA%E8%BD%AF%E6%9E%A3%E7%8C%95%E7%8C%B4%E6%A1%83%E8%BD%AC%E5%BD%95%E7%BB%84%E6%B5%8B%E5%BA%8F%E6%8A%80%E6%9C%AF%E6%9C%8D%E5%8A%A1%E6%8A%A5%E5%91%8A_20211126\\MWXS-21-2190D\\06.Differential_analysis\\Lc2_vs_Qssg\\pathway_enrichment\\maps\\ko00940.html) | ko00940 | 0.000000002 |
| [Plant-pathogen interaction](D:\\%E7%8E%8B%E7%A6%B9%E5%8D%9A\\%E7%A6%B9%E5%8D%9A%E5%8D%9A%E5%A3%AB%E5%8F%91%E8%A1%A8%E6%96%87%E7%AB%A0\\%E5%8F%91%E8%A1%A8SCI%E6%96%87%E7%AB%A0\\2021\\%E6%80%BB%E5%8C%96%E5%90%88%E7%89%A9%E8%BD%AC%E5%BD%95%E4%BB%A3%E8%B0%A2%E5%8F%8A%E8%81%94%E5%90%88%E5%88%86%E6%9E%90\\MWXS-21-2190D_%E8%BE%BD%E5%AE%81%E7%9C%81%E7%A7%91%E6%8A%80%E5%A4%A7%E5%AD%A66%E4%B8%AA%E8%BD%AF%E6%9E%A3%E7%8C%95%E7%8C%B4%E6%A1%83%E8%BD%AC%E5%BD%95%E7%BB%84%E6%B5%8B%E5%BA%8F%E6%8A%80%E6%9C%AF%E6%9C%8D%E5%8A%A1%E6%8A%A5%E5%91%8A_20211126\\MWXS-21-2190D\\06.Differential_analysis\\Lc2_vs_Qssg\\pathway_enrichment\\maps\\ko04626.html) | ko04626 | 0.000000054 |
| [Sesquiterpenoid and triterpenoid biosynthesis](D:\\%E7%8E%8B%E7%A6%B9%E5%8D%9A\\%E7%A6%B9%E5%8D%9A%E5%8D%9A%E5%A3%AB%E5%8F%91%E8%A1%A8%E6%96%87%E7%AB%A0\\%E5%8F%91%E8%A1%A8SCI%E6%96%87%E7%AB%A0\\2021\\%E6%80%BB%E5%8C%96%E5%90%88%E7%89%A9%E8%BD%AC%E5%BD%95%E4%BB%A3%E8%B0%A2%E5%8F%8A%E8%81%94%E5%90%88%E5%88%86%E6%9E%90\\MWXS-21-2190D_%E8%BE%BD%E5%AE%81%E7%9C%81%E7%A7%91%E6%8A%80%E5%A4%A7%E5%AD%A66%E4%B8%AA%E8%BD%AF%E6%9E%A3%E7%8C%95%E7%8C%B4%E6%A1%83%E8%BD%AC%E5%BD%95%E7%BB%84%E6%B5%8B%E5%BA%8F%E6%8A%80%E6%9C%AF%E6%9C%8D%E5%8A%A1%E6%8A%A5%E5%91%8A_20211126\\MWXS-21-2190D\\06.Differential_analysis\\Lc2_vs_Qssg\\pathway_enrichment\\maps\\ko00909.html) | ko00909 | 0.000000067 |
| [Cyanoamino acid metabolism](D:\\%E7%8E%8B%E7%A6%B9%E5%8D%9A\\%E7%A6%B9%E5%8D%9A%E5%8D%9A%E5%A3%AB%E5%8F%91%E8%A1%A8%E6%96%87%E7%AB%A0\\%E5%8F%91%E8%A1%A8SCI%E6%96%87%E7%AB%A0\\2021\\%E6%80%BB%E5%8C%96%E5%90%88%E7%89%A9%E8%BD%AC%E5%BD%95%E4%BB%A3%E8%B0%A2%E5%8F%8A%E8%81%94%E5%90%88%E5%88%86%E6%9E%90\\MWXS-21-2190D_%E8%BE%BD%E5%AE%81%E7%9C%81%E7%A7%91%E6%8A%80%E5%A4%A7%E5%AD%A66%E4%B8%AA%E8%BD%AF%E6%9E%A3%E7%8C%95%E7%8C%B4%E6%A1%83%E8%BD%AC%E5%BD%95%E7%BB%84%E6%B5%8B%E5%BA%8F%E6%8A%80%E6%9C%AF%E6%9C%8D%E5%8A%A1%E6%8A%A5%E5%91%8A_20211126\\MWXS-21-2190D\\06.Differential_analysis\\Lc2_vs_Qssg\\pathway_enrichment\\maps\\ko00460.html) | ko00460 | 0.000033405 |
| [Cysteine and methionine metabolism](D:\\%E7%8E%8B%E7%A6%B9%E5%8D%9A\\%E7%A6%B9%E5%8D%9A%E5%8D%9A%E5%A3%AB%E5%8F%91%E8%A1%A8%E6%96%87%E7%AB%A0\\%E5%8F%91%E8%A1%A8SCI%E6%96%87%E7%AB%A0\\2021\\%E6%80%BB%E5%8C%96%E5%90%88%E7%89%A9%E8%BD%AC%E5%BD%95%E4%BB%A3%E8%B0%A2%E5%8F%8A%E8%81%94%E5%90%88%E5%88%86%E6%9E%90\\MWXS-21-2190D_%E8%BE%BD%E5%AE%81%E7%9C%81%E7%A7%91%E6%8A%80%E5%A4%A7%E5%AD%A66%E4%B8%AA%E8%BD%AF%E6%9E%A3%E7%8C%95%E7%8C%B4%E6%A1%83%E8%BD%AC%E5%BD%95%E7%BB%84%E6%B5%8B%E5%BA%8F%E6%8A%80%E6%9C%AF%E6%9C%8D%E5%8A%A1%E6%8A%A5%E5%91%8A_20211126\\MWXS-21-2190D\\06.Differential_analysis\\Lc2_vs_Qssg\\pathway_enrichment\\maps\\ko00270.html) | ko00270 | 0.000035482 |
| [Glycosaminoglycan degradation](D:\\%E7%8E%8B%E7%A6%B9%E5%8D%9A\\%E7%A6%B9%E5%8D%9A%E5%8D%9A%E5%A3%AB%E5%8F%91%E8%A1%A8%E6%96%87%E7%AB%A0\\%E5%8F%91%E8%A1%A8SCI%E6%96%87%E7%AB%A0\\2021\\%E6%80%BB%E5%8C%96%E5%90%88%E7%89%A9%E8%BD%AC%E5%BD%95%E4%BB%A3%E8%B0%A2%E5%8F%8A%E8%81%94%E5%90%88%E5%88%86%E6%9E%90\\MWXS-21-2190D_%E8%BE%BD%E5%AE%81%E7%9C%81%E7%A7%91%E6%8A%80%E5%A4%A7%E5%AD%A66%E4%B8%AA%E8%BD%AF%E6%9E%A3%E7%8C%95%E7%8C%B4%E6%A1%83%E8%BD%AC%E5%BD%95%E7%BB%84%E6%B5%8B%E5%BA%8F%E6%8A%80%E6%9C%AF%E6%9C%8D%E5%8A%A1%E6%8A%A5%E5%91%8A_20211126\\MWXS-21-2190D\\06.Differential_analysis\\Lc2_vs_Qssg\\pathway_enrichment\\maps\\ko00531.html) | ko00531 | 0.000876133 |
| [Glycosphingolipid biosynthesis - ganglio series](D:\\%E7%8E%8B%E7%A6%B9%E5%8D%9A\\%E7%A6%B9%E5%8D%9A%E5%8D%9A%E5%A3%AB%E5%8F%91%E8%A1%A8%E6%96%87%E7%AB%A0\\%E5%8F%91%E8%A1%A8SCI%E6%96%87%E7%AB%A0\\2021\\%E6%80%BB%E5%8C%96%E5%90%88%E7%89%A9%E8%BD%AC%E5%BD%95%E4%BB%A3%E8%B0%A2%E5%8F%8A%E8%81%94%E5%90%88%E5%88%86%E6%9E%90\\MWXS-21-2190D_%E8%BE%BD%E5%AE%81%E7%9C%81%E7%A7%91%E6%8A%80%E5%A4%A7%E5%AD%A66%E4%B8%AA%E8%BD%AF%E6%9E%A3%E7%8C%95%E7%8C%B4%E6%A1%83%E8%BD%AC%E5%BD%95%E7%BB%84%E6%B5%8B%E5%BA%8F%E6%8A%80%E6%9C%AF%E6%9C%8D%E5%8A%A1%E6%8A%A5%E5%91%8A_20211126\\MWXS-21-2190D\\06.Differential_analysis\\Lc2_vs_Qssg\\pathway_enrichment\\maps\\ko00604.html) | ko00604 | 0.001037804 |
| [alpha-Linolenic acid metabolism](D:\\%E7%8E%8B%E7%A6%B9%E5%8D%9A\\%E7%A6%B9%E5%8D%9A%E5%8D%9A%E5%A3%AB%E5%8F%91%E8%A1%A8%E6%96%87%E7%AB%A0\\%E5%8F%91%E8%A1%A8SCI%E6%96%87%E7%AB%A0\\2021\\%E6%80%BB%E5%8C%96%E5%90%88%E7%89%A9%E8%BD%AC%E5%BD%95%E4%BB%A3%E8%B0%A2%E5%8F%8A%E8%81%94%E5%90%88%E5%88%86%E6%9E%90\\MWXS-21-2190D_%E8%BE%BD%E5%AE%81%E7%9C%81%E7%A7%91%E6%8A%80%E5%A4%A7%E5%AD%A66%E4%B8%AA%E8%BD%AF%E6%9E%A3%E7%8C%95%E7%8C%B4%E6%A1%83%E8%BD%AC%E5%BD%95%E7%BB%84%E6%B5%8B%E5%BA%8F%E6%8A%80%E6%9C%AF%E6%9C%8D%E5%8A%A1%E6%8A%A5%E5%91%8A_20211126\\MWXS-21-2190D\\06.Differential_analysis\\Lc2_vs_Qssg\\pathway_enrichment\\maps\\ko00592.html) | ko00592 | 0.001205077 |
| [Terpenoid backbone biosynthesis](D:\\%E7%8E%8B%E7%A6%B9%E5%8D%9A\\%E7%A6%B9%E5%8D%9A%E5%8D%9A%E5%A3%AB%E5%8F%91%E8%A1%A8%E6%96%87%E7%AB%A0\\%E5%8F%91%E8%A1%A8SCI%E6%96%87%E7%AB%A0\\2021\\%E6%80%BB%E5%8C%96%E5%90%88%E7%89%A9%E8%BD%AC%E5%BD%95%E4%BB%A3%E8%B0%A2%E5%8F%8A%E8%81%94%E5%90%88%E5%88%86%E6%9E%90\\MWXS-21-2190D_%E8%BE%BD%E5%AE%81%E7%9C%81%E7%A7%91%E6%8A%80%E5%A4%A7%E5%AD%A66%E4%B8%AA%E8%BD%AF%E6%9E%A3%E7%8C%95%E7%8C%B4%E6%A1%83%E8%BD%AC%E5%BD%95%E7%BB%84%E6%B5%8B%E5%BA%8F%E6%8A%80%E6%9C%AF%E6%9C%8D%E5%8A%A1%E6%8A%A5%E5%91%8A_20211126\\MWXS-21-2190D\\06.Differential_analysis\\Lc2_vs_Qssg\\pathway_enrichment\\maps\\ko00900.html) | ko00900 | 0.001584722 |
| [Flavonoid biosynthesis](D:\\%E7%8E%8B%E7%A6%B9%E5%8D%9A\\%E7%A6%B9%E5%8D%9A%E5%8D%9A%E5%A3%AB%E5%8F%91%E8%A1%A8%E6%96%87%E7%AB%A0\\%E5%8F%91%E8%A1%A8SCI%E6%96%87%E7%AB%A0\\2021\\%E6%80%BB%E5%8C%96%E5%90%88%E7%89%A9%E8%BD%AC%E5%BD%95%E4%BB%A3%E8%B0%A2%E5%8F%8A%E8%81%94%E5%90%88%E5%88%86%E6%9E%90\\MWXS-21-2190D_%E8%BE%BD%E5%AE%81%E7%9C%81%E7%A7%91%E6%8A%80%E5%A4%A7%E5%AD%A66%E4%B8%AA%E8%BD%AF%E6%9E%A3%E7%8C%95%E7%8C%B4%E6%A1%83%E8%BD%AC%E5%BD%95%E7%BB%84%E6%B5%8B%E5%BA%8F%E6%8A%80%E6%9C%AF%E6%9C%8D%E5%8A%A1%E6%8A%A5%E5%91%8A_20211126\\MWXS-21-2190D\\06.Differential_analysis\\Lc2_vs_Qssg\\pathway_enrichment\\maps\\ko00941.html) | ko00941 | 0.001635585 |
| [Cutin, suberine and wax biosynthesis](D:\\%E7%8E%8B%E7%A6%B9%E5%8D%9A\\%E7%A6%B9%E5%8D%9A%E5%8D%9A%E5%A3%AB%E5%8F%91%E8%A1%A8%E6%96%87%E7%AB%A0\\%E5%8F%91%E8%A1%A8SCI%E6%96%87%E7%AB%A0\\2021\\%E6%80%BB%E5%8C%96%E5%90%88%E7%89%A9%E8%BD%AC%E5%BD%95%E4%BB%A3%E8%B0%A2%E5%8F%8A%E8%81%94%E5%90%88%E5%88%86%E6%9E%90\\MWXS-21-2190D_%E8%BE%BD%E5%AE%81%E7%9C%81%E7%A7%91%E6%8A%80%E5%A4%A7%E5%AD%A66%E4%B8%AA%E8%BD%AF%E6%9E%A3%E7%8C%95%E7%8C%B4%E6%A1%83%E8%BD%AC%E5%BD%95%E7%BB%84%E6%B5%8B%E5%BA%8F%E6%8A%80%E6%9C%AF%E6%9C%8D%E5%8A%A1%E6%8A%A5%E5%91%8A_20211126\\MWXS-21-2190D\\06.Differential_analysis\\Lc2_vs_Qssg\\pathway_enrichment\\maps\\ko00073.html) | ko00073 | 0.001657583 |
| [Synthesis and degradation of ketone bodies](D:\\%E7%8E%8B%E7%A6%B9%E5%8D%9A\\%E7%A6%B9%E5%8D%9A%E5%8D%9A%E5%A3%AB%E5%8F%91%E8%A1%A8%E6%96%87%E7%AB%A0\\%E5%8F%91%E8%A1%A8SCI%E6%96%87%E7%AB%A0\\2021\\%E6%80%BB%E5%8C%96%E5%90%88%E7%89%A9%E8%BD%AC%E5%BD%95%E4%BB%A3%E8%B0%A2%E5%8F%8A%E8%81%94%E5%90%88%E5%88%86%E6%9E%90\\MWXS-21-2190D_%E8%BE%BD%E5%AE%81%E7%9C%81%E7%A7%91%E6%8A%80%E5%A4%A7%E5%AD%A66%E4%B8%AA%E8%BD%AF%E6%9E%A3%E7%8C%95%E7%8C%B4%E6%A1%83%E8%BD%AC%E5%BD%95%E7%BB%84%E6%B5%8B%E5%BA%8F%E6%8A%80%E6%9C%AF%E6%9C%8D%E5%8A%A1%E6%8A%A5%E5%91%8A_20211126\\MWXS-21-2190D\\06.Differential_analysis\\Lc2_vs_Qssg\\pathway_enrichment\\maps\\ko00072.html) | ko00072 | 0.001689141 |
| [Diterpenoid biosynthesis](D:\\%E7%8E%8B%E7%A6%B9%E5%8D%9A\\%E7%A6%B9%E5%8D%9A%E5%8D%9A%E5%A3%AB%E5%8F%91%E8%A1%A8%E6%96%87%E7%AB%A0\\%E5%8F%91%E8%A1%A8SCI%E6%96%87%E7%AB%A0\\2021\\%E6%80%BB%E5%8C%96%E5%90%88%E7%89%A9%E8%BD%AC%E5%BD%95%E4%BB%A3%E8%B0%A2%E5%8F%8A%E8%81%94%E5%90%88%E5%88%86%E6%9E%90\\MWXS-21-2190D_%E8%BE%BD%E5%AE%81%E7%9C%81%E7%A7%91%E6%8A%80%E5%A4%A7%E5%AD%A66%E4%B8%AA%E8%BD%AF%E6%9E%A3%E7%8C%95%E7%8C%B4%E6%A1%83%E8%BD%AC%E5%BD%95%E7%BB%84%E6%B5%8B%E5%BA%8F%E6%8A%80%E6%9C%AF%E6%9C%8D%E5%8A%A1%E6%8A%A5%E5%91%8A_20211126\\MWXS-21-2190D\\06.Differential_analysis\\Lc2_vs_Qssg\\pathway_enrichment\\maps\\ko00904.html) | ko00904 | 0.002002703 |
| [Butanoate metabolism](D:\\%E7%8E%8B%E7%A6%B9%E5%8D%9A\\%E7%A6%B9%E5%8D%9A%E5%8D%9A%E5%A3%AB%E5%8F%91%E8%A1%A8%E6%96%87%E7%AB%A0\\%E5%8F%91%E8%A1%A8SCI%E6%96%87%E7%AB%A0\\2021\\%E6%80%BB%E5%8C%96%E5%90%88%E7%89%A9%E8%BD%AC%E5%BD%95%E4%BB%A3%E8%B0%A2%E5%8F%8A%E8%81%94%E5%90%88%E5%88%86%E6%9E%90\\MWXS-21-2190D_%E8%BE%BD%E5%AE%81%E7%9C%81%E7%A7%91%E6%8A%80%E5%A4%A7%E5%AD%A66%E4%B8%AA%E8%BD%AF%E6%9E%A3%E7%8C%95%E7%8C%B4%E6%A1%83%E8%BD%AC%E5%BD%95%E7%BB%84%E6%B5%8B%E5%BA%8F%E6%8A%80%E6%9C%AF%E6%9C%8D%E5%8A%A1%E6%8A%A5%E5%91%8A_20211126\\MWXS-21-2190D\\06.Differential_analysis\\Lc2_vs_Qssg\\pathway_enrichment\\maps\\ko00650.html) | ko00650 | 0.003666692 |
| [Monoterpenoid biosynthesis](D:\\%E7%8E%8B%E7%A6%B9%E5%8D%9A\\%E7%A6%B9%E5%8D%9A%E5%8D%9A%E5%A3%AB%E5%8F%91%E8%A1%A8%E6%96%87%E7%AB%A0\\%E5%8F%91%E8%A1%A8SCI%E6%96%87%E7%AB%A0\\2021\\%E6%80%BB%E5%8C%96%E5%90%88%E7%89%A9%E8%BD%AC%E5%BD%95%E4%BB%A3%E8%B0%A2%E5%8F%8A%E8%81%94%E5%90%88%E5%88%86%E6%9E%90\\MWXS-21-2190D_%E8%BE%BD%E5%AE%81%E7%9C%81%E7%A7%91%E6%8A%80%E5%A4%A7%E5%AD%A66%E4%B8%AA%E8%BD%AF%E6%9E%A3%E7%8C%95%E7%8C%B4%E6%A1%83%E8%BD%AC%E5%BD%95%E7%BB%84%E6%B5%8B%E5%BA%8F%E6%8A%80%E6%9C%AF%E6%9C%8D%E5%8A%A1%E6%8A%A5%E5%91%8A_20211126\\MWXS-21-2190D\\06.Differential_analysis\\Lc2_vs_Qssg\\pathway_enrichment\\maps\\ko00902.html) | ko00902 | 0.003808836 |
| [Pentose and glucuronate interconversions](D:\\%E7%8E%8B%E7%A6%B9%E5%8D%9A\\%E7%A6%B9%E5%8D%9A%E5%8D%9A%E5%A3%AB%E5%8F%91%E8%A1%A8%E6%96%87%E7%AB%A0\\%E5%8F%91%E8%A1%A8SCI%E6%96%87%E7%AB%A0\\2021\\%E6%80%BB%E5%8C%96%E5%90%88%E7%89%A9%E8%BD%AC%E5%BD%95%E4%BB%A3%E8%B0%A2%E5%8F%8A%E8%81%94%E5%90%88%E5%88%86%E6%9E%90\\MWXS-21-2190D_%E8%BE%BD%E5%AE%81%E7%9C%81%E7%A7%91%E6%8A%80%E5%A4%A7%E5%AD%A66%E4%B8%AA%E8%BD%AF%E6%9E%A3%E7%8C%95%E7%8C%B4%E6%A1%83%E8%BD%AC%E5%BD%95%E7%BB%84%E6%B5%8B%E5%BA%8F%E6%8A%80%E6%9C%AF%E6%9C%8D%E5%8A%A1%E6%8A%A5%E5%91%8A_20211126\\MWXS-21-2190D\\06.Differential_analysis\\Lc2_vs_Qssg\\pathway_enrichment\\maps\\ko00040.html) | ko00040 | 0.005000041 |
| [Stilbenoid, diarylheptanoid and gingerol biosynthesis](D:\\%E7%8E%8B%E7%A6%B9%E5%8D%9A\\%E7%A6%B9%E5%8D%9A%E5%8D%9A%E5%A3%AB%E5%8F%91%E8%A1%A8%E6%96%87%E7%AB%A0\\%E5%8F%91%E8%A1%A8SCI%E6%96%87%E7%AB%A0\\2021\\%E6%80%BB%E5%8C%96%E5%90%88%E7%89%A9%E8%BD%AC%E5%BD%95%E4%BB%A3%E8%B0%A2%E5%8F%8A%E8%81%94%E5%90%88%E5%88%86%E6%9E%90\\MWXS-21-2190D_%E8%BE%BD%E5%AE%81%E7%9C%81%E7%A7%91%E6%8A%80%E5%A4%A7%E5%AD%A66%E4%B8%AA%E8%BD%AF%E6%9E%A3%E7%8C%95%E7%8C%B4%E6%A1%83%E8%BD%AC%E5%BD%95%E7%BB%84%E6%B5%8B%E5%BA%8F%E6%8A%80%E6%9C%AF%E6%9C%8D%E5%8A%A1%E6%8A%A5%E5%91%8A_20211126\\MWXS-21-2190D\\06.Differential_analysis\\Lc2_vs_Qssg\\pathway_enrichment\\maps\\ko00945.html) | ko00945 | 0.005054709 |
| [Alanine, aspartate and glutamate metabolism](D:\\%E7%8E%8B%E7%A6%B9%E5%8D%9A\\%E7%A6%B9%E5%8D%9A%E5%8D%9A%E5%A3%AB%E5%8F%91%E8%A1%A8%E6%96%87%E7%AB%A0\\%E5%8F%91%E8%A1%A8SCI%E6%96%87%E7%AB%A0\\2021\\%E6%80%BB%E5%8C%96%E5%90%88%E7%89%A9%E8%BD%AC%E5%BD%95%E4%BB%A3%E8%B0%A2%E5%8F%8A%E8%81%94%E5%90%88%E5%88%86%E6%9E%90\\MWXS-21-2190D_%E8%BE%BD%E5%AE%81%E7%9C%81%E7%A7%91%E6%8A%80%E5%A4%A7%E5%AD%A66%E4%B8%AA%E8%BD%AF%E6%9E%A3%E7%8C%95%E7%8C%B4%E6%A1%83%E8%BD%AC%E5%BD%95%E7%BB%84%E6%B5%8B%E5%BA%8F%E6%8A%80%E6%9C%AF%E6%9C%8D%E5%8A%A1%E6%8A%A5%E5%91%8A_20211126\\MWXS-21-2190D\\06.Differential_analysis\\Lc2_vs_Qssg\\pathway_enrichment\\maps\\ko00250.html) | ko00250 | 0.006093694 |
| [Other glycan degradation](D:\\%E7%8E%8B%E7%A6%B9%E5%8D%9A\\%E7%A6%B9%E5%8D%9A%E5%8D%9A%E5%A3%AB%E5%8F%91%E8%A1%A8%E6%96%87%E7%AB%A0\\%E5%8F%91%E8%A1%A8SCI%E6%96%87%E7%AB%A0\\2021\\%E6%80%BB%E5%8C%96%E5%90%88%E7%89%A9%E8%BD%AC%E5%BD%95%E4%BB%A3%E8%B0%A2%E5%8F%8A%E8%81%94%E5%90%88%E5%88%86%E6%9E%90\\MWXS-21-2190D_%E8%BE%BD%E5%AE%81%E7%9C%81%E7%A7%91%E6%8A%80%E5%A4%A7%E5%AD%A66%E4%B8%AA%E8%BD%AF%E6%9E%A3%E7%8C%95%E7%8C%B4%E6%A1%83%E8%BD%AC%E5%BD%95%E7%BB%84%E6%B5%8B%E5%BA%8F%E6%8A%80%E6%9C%AF%E6%9C%8D%E5%8A%A1%E6%8A%A5%E5%91%8A_20211126\\MWXS-21-2190D\\06.Differential_analysis\\Lc2_vs_Qssg\\pathway_enrichment\\maps\\ko00511.html) | ko00511 | 0.007389909 |
| [Valine, leucine and isoleucine degradation](D:\\%E7%8E%8B%E7%A6%B9%E5%8D%9A\\%E7%A6%B9%E5%8D%9A%E5%8D%9A%E5%A3%AB%E5%8F%91%E8%A1%A8%E6%96%87%E7%AB%A0\\%E5%8F%91%E8%A1%A8SCI%E6%96%87%E7%AB%A0\\2021\\%E6%80%BB%E5%8C%96%E5%90%88%E7%89%A9%E8%BD%AC%E5%BD%95%E4%BB%A3%E8%B0%A2%E5%8F%8A%E8%81%94%E5%90%88%E5%88%86%E6%9E%90\\MWXS-21-2190D_%E8%BE%BD%E5%AE%81%E7%9C%81%E7%A7%91%E6%8A%80%E5%A4%A7%E5%AD%A66%E4%B8%AA%E8%BD%AF%E6%9E%A3%E7%8C%95%E7%8C%B4%E6%A1%83%E8%BD%AC%E5%BD%95%E7%BB%84%E6%B5%8B%E5%BA%8F%E6%8A%80%E6%9C%AF%E6%9C%8D%E5%8A%A1%E6%8A%A5%E5%91%8A_20211126\\MWXS-21-2190D\\06.Differential_analysis\\Lc2_vs_Qssg\\pathway_enrichment\\maps\\ko00280.html) | ko00280 | 0.007785754 |
| [Glutathione metabolism](D:\\%E7%8E%8B%E7%A6%B9%E5%8D%9A\\%E7%A6%B9%E5%8D%9A%E5%8D%9A%E5%A3%AB%E5%8F%91%E8%A1%A8%E6%96%87%E7%AB%A0\\%E5%8F%91%E8%A1%A8SCI%E6%96%87%E7%AB%A0\\2021\\%E6%80%BB%E5%8C%96%E5%90%88%E7%89%A9%E8%BD%AC%E5%BD%95%E4%BB%A3%E8%B0%A2%E5%8F%8A%E8%81%94%E5%90%88%E5%88%86%E6%9E%90\\MWXS-21-2190D_%E8%BE%BD%E5%AE%81%E7%9C%81%E7%A7%91%E6%8A%80%E5%A4%A7%E5%AD%A66%E4%B8%AA%E8%BD%AF%E6%9E%A3%E7%8C%95%E7%8C%B4%E6%A1%83%E8%BD%AC%E5%BD%95%E7%BB%84%E6%B5%8B%E5%BA%8F%E6%8A%80%E6%9C%AF%E6%9C%8D%E5%8A%A1%E6%8A%A5%E5%91%8A_20211126\\MWXS-21-2190D\\06.Differential_analysis\\Lc2_vs_Qssg\\pathway_enrichment\\maps\\ko00480.html) | ko00480 | 0.008340556 |
| [Phenylalanine, tyrosine and tryptophan biosynthesis](D:\\%E7%8E%8B%E7%A6%B9%E5%8D%9A\\%E7%A6%B9%E5%8D%9A%E5%8D%9A%E5%A3%AB%E5%8F%91%E8%A1%A8%E6%96%87%E7%AB%A0\\%E5%8F%91%E8%A1%A8SCI%E6%96%87%E7%AB%A0\\2021\\%E6%80%BB%E5%8C%96%E5%90%88%E7%89%A9%E8%BD%AC%E5%BD%95%E4%BB%A3%E8%B0%A2%E5%8F%8A%E8%81%94%E5%90%88%E5%88%86%E6%9E%90\\MWXS-21-2190D_%E8%BE%BD%E5%AE%81%E7%9C%81%E7%A7%91%E6%8A%80%E5%A4%A7%E5%AD%A66%E4%B8%AA%E8%BD%AF%E6%9E%A3%E7%8C%95%E7%8C%B4%E6%A1%83%E8%BD%AC%E5%BD%95%E7%BB%84%E6%B5%8B%E5%BA%8F%E6%8A%80%E6%9C%AF%E6%9C%8D%E5%8A%A1%E6%8A%A5%E5%91%8A_20211126\\MWXS-21-2190D\\06.Differential_analysis\\Lc2_vs_Qssg\\pathway_enrichment\\maps\\ko00400.html) | ko00400 | 0.008765574 |
| [Linoleic acid metabolism](D:\\%E7%8E%8B%E7%A6%B9%E5%8D%9A\\%E7%A6%B9%E5%8D%9A%E5%8D%9A%E5%A3%AB%E5%8F%91%E8%A1%A8%E6%96%87%E7%AB%A0\\%E5%8F%91%E8%A1%A8SCI%E6%96%87%E7%AB%A0\\2021\\%E6%80%BB%E5%8C%96%E5%90%88%E7%89%A9%E8%BD%AC%E5%BD%95%E4%BB%A3%E8%B0%A2%E5%8F%8A%E8%81%94%E5%90%88%E5%88%86%E6%9E%90\\MWXS-21-2190D_%E8%BE%BD%E5%AE%81%E7%9C%81%E7%A7%91%E6%8A%80%E5%A4%A7%E5%AD%A66%E4%B8%AA%E8%BD%AF%E6%9E%A3%E7%8C%95%E7%8C%B4%E6%A1%83%E8%BD%AC%E5%BD%95%E7%BB%84%E6%B5%8B%E5%BA%8F%E6%8A%80%E6%9C%AF%E6%9C%8D%E5%8A%A1%E6%8A%A5%E5%91%8A_20211126\\MWXS-21-2190D\\06.Differential_analysis\\Lc2_vs_Qssg\\pathway_enrichment\\maps\\ko00591.html) | ko00591 | 0.013222159 |
| [Photosynthesis - antenna proteins](D:\\%E7%8E%8B%E7%A6%B9%E5%8D%9A\\%E7%A6%B9%E5%8D%9A%E5%8D%9A%E5%A3%AB%E5%8F%91%E8%A1%A8%E6%96%87%E7%AB%A0\\%E5%8F%91%E8%A1%A8SCI%E6%96%87%E7%AB%A0\\2021\\%E6%80%BB%E5%8C%96%E5%90%88%E7%89%A9%E8%BD%AC%E5%BD%95%E4%BB%A3%E8%B0%A2%E5%8F%8A%E8%81%94%E5%90%88%E5%88%86%E6%9E%90\\MWXS-21-2190D_%E8%BE%BD%E5%AE%81%E7%9C%81%E7%A7%91%E6%8A%80%E5%A4%A7%E5%AD%A66%E4%B8%AA%E8%BD%AF%E6%9E%A3%E7%8C%95%E7%8C%B4%E6%A1%83%E8%BD%AC%E5%BD%95%E7%BB%84%E6%B5%8B%E5%BA%8F%E6%8A%80%E6%9C%AF%E6%9C%8D%E5%8A%A1%E6%8A%A5%E5%91%8A_20211126\\MWXS-21-2190D\\06.Differential_analysis\\Lc2_vs_Qssg\\pathway_enrichment\\maps\\ko00196.html) | ko00196 | 0.013829013 |
| [Glucosinolate biosynthesis](D:\\%E7%8E%8B%E7%A6%B9%E5%8D%9A\\%E7%A6%B9%E5%8D%9A%E5%8D%9A%E5%A3%AB%E5%8F%91%E8%A1%A8%E6%96%87%E7%AB%A0\\%E5%8F%91%E8%A1%A8SCI%E6%96%87%E7%AB%A0\\2021\\%E6%80%BB%E5%8C%96%E5%90%88%E7%89%A9%E8%BD%AC%E5%BD%95%E4%BB%A3%E8%B0%A2%E5%8F%8A%E8%81%94%E5%90%88%E5%88%86%E6%9E%90\\MWXS-21-2190D_%E8%BE%BD%E5%AE%81%E7%9C%81%E7%A7%91%E6%8A%80%E5%A4%A7%E5%AD%A66%E4%B8%AA%E8%BD%AF%E6%9E%A3%E7%8C%95%E7%8C%B4%E6%A1%83%E8%BD%AC%E5%BD%95%E7%BB%84%E6%B5%8B%E5%BA%8F%E6%8A%80%E6%9C%AF%E6%9C%8D%E5%8A%A1%E6%8A%A5%E5%91%8A_20211126\\MWXS-21-2190D\\06.Differential_analysis\\Lc2_vs_Qssg\\pathway_enrichment\\maps\\ko00966.html) | ko00966 | 0.01551566 |
| [Glycerolipid metabolism](D:\\%E7%8E%8B%E7%A6%B9%E5%8D%9A\\%E7%A6%B9%E5%8D%9A%E5%8D%9A%E5%A3%AB%E5%8F%91%E8%A1%A8%E6%96%87%E7%AB%A0\\%E5%8F%91%E8%A1%A8SCI%E6%96%87%E7%AB%A0\\2021\\%E6%80%BB%E5%8C%96%E5%90%88%E7%89%A9%E8%BD%AC%E5%BD%95%E4%BB%A3%E8%B0%A2%E5%8F%8A%E8%81%94%E5%90%88%E5%88%86%E6%9E%90\\MWXS-21-2190D_%E8%BE%BD%E5%AE%81%E7%9C%81%E7%A7%91%E6%8A%80%E5%A4%A7%E5%AD%A66%E4%B8%AA%E8%BD%AF%E6%9E%A3%E7%8C%95%E7%8C%B4%E6%A1%83%E8%BD%AC%E5%BD%95%E7%BB%84%E6%B5%8B%E5%BA%8F%E6%8A%80%E6%9C%AF%E6%9C%8D%E5%8A%A1%E6%8A%A5%E5%91%8A_20211126\\MWXS-21-2190D\\06.Differential_analysis\\Lc2_vs_Qssg\\pathway_enrichment\\maps\\ko00561.html) | ko00561 | 0.018540377 |
| [Selenocompound metabolism](D:\\%E7%8E%8B%E7%A6%B9%E5%8D%9A\\%E7%A6%B9%E5%8D%9A%E5%8D%9A%E5%A3%AB%E5%8F%91%E8%A1%A8%E6%96%87%E7%AB%A0\\%E5%8F%91%E8%A1%A8SCI%E6%96%87%E7%AB%A0\\2021\\%E6%80%BB%E5%8C%96%E5%90%88%E7%89%A9%E8%BD%AC%E5%BD%95%E4%BB%A3%E8%B0%A2%E5%8F%8A%E8%81%94%E5%90%88%E5%88%86%E6%9E%90\\MWXS-21-2190D_%E8%BE%BD%E5%AE%81%E7%9C%81%E7%A7%91%E6%8A%80%E5%A4%A7%E5%AD%A66%E4%B8%AA%E8%BD%AF%E6%9E%A3%E7%8C%95%E7%8C%B4%E6%A1%83%E8%BD%AC%E5%BD%95%E7%BB%84%E6%B5%8B%E5%BA%8F%E6%8A%80%E6%9C%AF%E6%9C%8D%E5%8A%A1%E6%8A%A5%E5%91%8A_20211126\\MWXS-21-2190D\\06.Differential_analysis\\Lc2_vs_Qssg\\pathway_enrichment\\maps\\ko00450.html) | ko00450 | 0.019562351 |
| [Arachidonic acid metabolism](D:\\%E7%8E%8B%E7%A6%B9%E5%8D%9A\\%E7%A6%B9%E5%8D%9A%E5%8D%9A%E5%A3%AB%E5%8F%91%E8%A1%A8%E6%96%87%E7%AB%A0\\%E5%8F%91%E8%A1%A8SCI%E6%96%87%E7%AB%A0\\2021\\%E6%80%BB%E5%8C%96%E5%90%88%E7%89%A9%E8%BD%AC%E5%BD%95%E4%BB%A3%E8%B0%A2%E5%8F%8A%E8%81%94%E5%90%88%E5%88%86%E6%9E%90\\MWXS-21-2190D_%E8%BE%BD%E5%AE%81%E7%9C%81%E7%A7%91%E6%8A%80%E5%A4%A7%E5%AD%A66%E4%B8%AA%E8%BD%AF%E6%9E%A3%E7%8C%95%E7%8C%B4%E6%A1%83%E8%BD%AC%E5%BD%95%E7%BB%84%E6%B5%8B%E5%BA%8F%E6%8A%80%E6%9C%AF%E6%9C%8D%E5%8A%A1%E6%8A%A5%E5%91%8A_20211126\\MWXS-21-2190D\\06.Differential_analysis\\Lc2_vs_Qssg\\pathway_enrichment\\maps\\ko00590.html) | ko00590 | 0.021050219 |
| [Isoquinoline alkaloid biosynthesis](D:\\%E7%8E%8B%E7%A6%B9%E5%8D%9A\\%E7%A6%B9%E5%8D%9A%E5%8D%9A%E5%A3%AB%E5%8F%91%E8%A1%A8%E6%96%87%E7%AB%A0\\%E5%8F%91%E8%A1%A8SCI%E6%96%87%E7%AB%A0\\2021\\%E6%80%BB%E5%8C%96%E5%90%88%E7%89%A9%E8%BD%AC%E5%BD%95%E4%BB%A3%E8%B0%A2%E5%8F%8A%E8%81%94%E5%90%88%E5%88%86%E6%9E%90\\MWXS-21-2190D_%E8%BE%BD%E5%AE%81%E7%9C%81%E7%A7%91%E6%8A%80%E5%A4%A7%E5%AD%A66%E4%B8%AA%E8%BD%AF%E6%9E%A3%E7%8C%95%E7%8C%B4%E6%A1%83%E8%BD%AC%E5%BD%95%E7%BB%84%E6%B5%8B%E5%BA%8F%E6%8A%80%E6%9C%AF%E6%9C%8D%E5%8A%A1%E6%8A%A5%E5%91%8A_20211126\\MWXS-21-2190D\\06.Differential_analysis\\Lc2_vs_Qssg\\pathway_enrichment\\maps\\ko00950.html) | ko00950 | 0.02211674 |
| [Glycerophospholipid metabolism](D:\\%E7%8E%8B%E7%A6%B9%E5%8D%9A\\%E7%A6%B9%E5%8D%9A%E5%8D%9A%E5%A3%AB%E5%8F%91%E8%A1%A8%E6%96%87%E7%AB%A0\\%E5%8F%91%E8%A1%A8SCI%E6%96%87%E7%AB%A0\\2021\\%E6%80%BB%E5%8C%96%E5%90%88%E7%89%A9%E8%BD%AC%E5%BD%95%E4%BB%A3%E8%B0%A2%E5%8F%8A%E8%81%94%E5%90%88%E5%88%86%E6%9E%90\\MWXS-21-2190D_%E8%BE%BD%E5%AE%81%E7%9C%81%E7%A7%91%E6%8A%80%E5%A4%A7%E5%AD%A66%E4%B8%AA%E8%BD%AF%E6%9E%A3%E7%8C%95%E7%8C%B4%E6%A1%83%E8%BD%AC%E5%BD%95%E7%BB%84%E6%B5%8B%E5%BA%8F%E6%8A%80%E6%9C%AF%E6%9C%8D%E5%8A%A1%E6%8A%A5%E5%91%8A_20211126\\MWXS-21-2190D\\06.Differential_analysis\\Lc2_vs_Qssg\\pathway_enrichment\\maps\\ko00564.html) | ko00564 | 0.023083063 |
| [Glyoxylate and dicarboxylate metabolism](D:\\%E7%8E%8B%E7%A6%B9%E5%8D%9A\\%E7%A6%B9%E5%8D%9A%E5%8D%9A%E5%A3%AB%E5%8F%91%E8%A1%A8%E6%96%87%E7%AB%A0\\%E5%8F%91%E8%A1%A8SCI%E6%96%87%E7%AB%A0\\2021\\%E6%80%BB%E5%8C%96%E5%90%88%E7%89%A9%E8%BD%AC%E5%BD%95%E4%BB%A3%E8%B0%A2%E5%8F%8A%E8%81%94%E5%90%88%E5%88%86%E6%9E%90\\MWXS-21-2190D_%E8%BE%BD%E5%AE%81%E7%9C%81%E7%A7%91%E6%8A%80%E5%A4%A7%E5%AD%A66%E4%B8%AA%E8%BD%AF%E6%9E%A3%E7%8C%95%E7%8C%B4%E6%A1%83%E8%BD%AC%E5%BD%95%E7%BB%84%E6%B5%8B%E5%BA%8F%E6%8A%80%E6%9C%AF%E6%9C%8D%E5%8A%A1%E6%8A%A5%E5%91%8A_20211126\\MWXS-21-2190D\\06.Differential_analysis\\Lc2_vs_Qssg\\pathway_enrichment\\maps\\ko00630.html) | ko00630 | 0.024573002 |
| [Tropane, piperidine and pyridine alkaloid biosynthesis](D:\\%E7%8E%8B%E7%A6%B9%E5%8D%9A\\%E7%A6%B9%E5%8D%9A%E5%8D%9A%E5%A3%AB%E5%8F%91%E8%A1%A8%E6%96%87%E7%AB%A0\\%E5%8F%91%E8%A1%A8SCI%E6%96%87%E7%AB%A0\\2021\\%E6%80%BB%E5%8C%96%E5%90%88%E7%89%A9%E8%BD%AC%E5%BD%95%E4%BB%A3%E8%B0%A2%E5%8F%8A%E8%81%94%E5%90%88%E5%88%86%E6%9E%90\\MWXS-21-2190D_%E8%BE%BD%E5%AE%81%E7%9C%81%E7%A7%91%E6%8A%80%E5%A4%A7%E5%AD%A66%E4%B8%AA%E8%BD%AF%E6%9E%A3%E7%8C%95%E7%8C%B4%E6%A1%83%E8%BD%AC%E5%BD%95%E7%BB%84%E6%B5%8B%E5%BA%8F%E6%8A%80%E6%9C%AF%E6%9C%8D%E5%8A%A1%E6%8A%A5%E5%91%8A_20211126\\MWXS-21-2190D\\06.Differential_analysis\\Lc2_vs_Qssg\\pathway_enrichment\\maps\\ko00960.html) | ko00960 | 0.026807536 |
| [Isoflavonoid biosynthesis](D:\\%E7%8E%8B%E7%A6%B9%E5%8D%9A\\%E7%A6%B9%E5%8D%9A%E5%8D%9A%E5%A3%AB%E5%8F%91%E8%A1%A8%E6%96%87%E7%AB%A0\\%E5%8F%91%E8%A1%A8SCI%E6%96%87%E7%AB%A0\\2021\\%E6%80%BB%E5%8C%96%E5%90%88%E7%89%A9%E8%BD%AC%E5%BD%95%E4%BB%A3%E8%B0%A2%E5%8F%8A%E8%81%94%E5%90%88%E5%88%86%E6%9E%90\\MWXS-21-2190D_%E8%BE%BD%E5%AE%81%E7%9C%81%E7%A7%91%E6%8A%80%E5%A4%A7%E5%AD%A66%E4%B8%AA%E8%BD%AF%E6%9E%A3%E7%8C%95%E7%8C%B4%E6%A1%83%E8%BD%AC%E5%BD%95%E7%BB%84%E6%B5%8B%E5%BA%8F%E6%8A%80%E6%9C%AF%E6%9C%8D%E5%8A%A1%E6%8A%A5%E5%91%8A_20211126\\MWXS-21-2190D\\06.Differential_analysis\\Lc2_vs_Qssg\\pathway_enrichment\\maps\\ko00943.html) | ko00943 | 0.026965742 |
| [Biosynthesis of unsaturated fatty acids](D:\\%E7%8E%8B%E7%A6%B9%E5%8D%9A\\%E7%A6%B9%E5%8D%9A%E5%8D%9A%E5%A3%AB%E5%8F%91%E8%A1%A8%E6%96%87%E7%AB%A0\\%E5%8F%91%E8%A1%A8SCI%E6%96%87%E7%AB%A0\\2021\\%E6%80%BB%E5%8C%96%E5%90%88%E7%89%A9%E8%BD%AC%E5%BD%95%E4%BB%A3%E8%B0%A2%E5%8F%8A%E8%81%94%E5%90%88%E5%88%86%E6%9E%90\\MWXS-21-2190D_%E8%BE%BD%E5%AE%81%E7%9C%81%E7%A7%91%E6%8A%80%E5%A4%A7%E5%AD%A66%E4%B8%AA%E8%BD%AF%E6%9E%A3%E7%8C%95%E7%8C%B4%E6%A1%83%E8%BD%AC%E5%BD%95%E7%BB%84%E6%B5%8B%E5%BA%8F%E6%8A%80%E6%9C%AF%E6%9C%8D%E5%8A%A1%E6%8A%A5%E5%91%8A_20211126\\MWXS-21-2190D\\06.Differential_analysis\\Lc2_vs_Qssg\\pathway_enrichment\\maps\\ko01040.html) | ko01040 | 0.030429475 |
| [Galactose metabolism](D:\\%E7%8E%8B%E7%A6%B9%E5%8D%9A\\%E7%A6%B9%E5%8D%9A%E5%8D%9A%E5%A3%AB%E5%8F%91%E8%A1%A8%E6%96%87%E7%AB%A0\\%E5%8F%91%E8%A1%A8SCI%E6%96%87%E7%AB%A0\\2021\\%E6%80%BB%E5%8C%96%E5%90%88%E7%89%A9%E8%BD%AC%E5%BD%95%E4%BB%A3%E8%B0%A2%E5%8F%8A%E8%81%94%E5%90%88%E5%88%86%E6%9E%90\\MWXS-21-2190D_%E8%BE%BD%E5%AE%81%E7%9C%81%E7%A7%91%E6%8A%80%E5%A4%A7%E5%AD%A66%E4%B8%AA%E8%BD%AF%E6%9E%A3%E7%8C%95%E7%8C%B4%E6%A1%83%E8%BD%AC%E5%BD%95%E7%BB%84%E6%B5%8B%E5%BA%8F%E6%8A%80%E6%9C%AF%E6%9C%8D%E5%8A%A1%E6%8A%A5%E5%91%8A_20211126\\MWXS-21-2190D\\06.Differential_analysis\\Lc2_vs_Qssg\\pathway_enrichment\\maps\\ko00052.html) | ko00052 | 0.037660903 |
| [Ether lipid metabolism](D:\\%E7%8E%8B%E7%A6%B9%E5%8D%9A\\%E7%A6%B9%E5%8D%9A%E5%8D%9A%E5%A3%AB%E5%8F%91%E8%A1%A8%E6%96%87%E7%AB%A0\\%E5%8F%91%E8%A1%A8SCI%E6%96%87%E7%AB%A0\\2021\\%E6%80%BB%E5%8C%96%E5%90%88%E7%89%A9%E8%BD%AC%E5%BD%95%E4%BB%A3%E8%B0%A2%E5%8F%8A%E8%81%94%E5%90%88%E5%88%86%E6%9E%90\\MWXS-21-2190D_%E8%BE%BD%E5%AE%81%E7%9C%81%E7%A7%91%E6%8A%80%E5%A4%A7%E5%AD%A66%E4%B8%AA%E8%BD%AF%E6%9E%A3%E7%8C%95%E7%8C%B4%E6%A1%83%E8%BD%AC%E5%BD%95%E7%BB%84%E6%B5%8B%E5%BA%8F%E6%8A%80%E6%9C%AF%E6%9C%8D%E5%8A%A1%E6%8A%A5%E5%91%8A_20211126\\MWXS-21-2190D\\06.Differential_analysis\\Lc2_vs_Qssg\\pathway_enrichment\\maps\\ko00565.html) | ko00565 | 0.041447636 |
| [Nitrogen metabolism](D:\\%E7%8E%8B%E7%A6%B9%E5%8D%9A\\%E7%A6%B9%E5%8D%9A%E5%8D%9A%E5%A3%AB%E5%8F%91%E8%A1%A8%E6%96%87%E7%AB%A0\\%E5%8F%91%E8%A1%A8SCI%E6%96%87%E7%AB%A0\\2021\\%E6%80%BB%E5%8C%96%E5%90%88%E7%89%A9%E8%BD%AC%E5%BD%95%E4%BB%A3%E8%B0%A2%E5%8F%8A%E8%81%94%E5%90%88%E5%88%86%E6%9E%90\\MWXS-21-2190D_%E8%BE%BD%E5%AE%81%E7%9C%81%E7%A7%91%E6%8A%80%E5%A4%A7%E5%AD%A66%E4%B8%AA%E8%BD%AF%E6%9E%A3%E7%8C%95%E7%8C%B4%E6%A1%83%E8%BD%AC%E5%BD%95%E7%BB%84%E6%B5%8B%E5%BA%8F%E6%8A%80%E6%9C%AF%E6%9C%8D%E5%8A%A1%E6%8A%A5%E5%91%8A_20211126\\MWXS-21-2190D\\06.Differential_analysis\\Lc2_vs_Qssg\\pathway_enrichment\\maps\\ko00910.html) | ko00910 | 0.047790018 |

| **Table S8. List of differential expression structural genes of flavonoid during**  Actinidia arguta fruit maturity | | | | |
| --- | --- | --- | --- | --- |
| **Gene  Name** | **Gene ID** | **Qssg vs Lc** | | |
|  |  | **FDR** | **Log2FC** | **up/down** |
| CsUGT134 | Cluster-10307.10035 | 7.84E-07 | 5.531302184 | up |
|  | Cluster-10307.5267 | 5.53E-44 | 13.18206007 | up |
|  | Cluster-10307.64523 | 7.98E-02 | 1.408579684 | up |
| LOC | Cluster-10307.11602 | 3.60E-03 | 1.812441233 | up |
|  | Cluster-10307.5031 | 1.60E-01 | 2.708097664 | up |
|  | Cluster-10307.5032 | 1.26E-02 | 2.847410903 | up |
|  | Cluster-10307.81571 | 3.52E-05 | 8.267330484 | up |
|  | Cluster-10307.81683 | 9.61E-02 | 1.662784198 | up |
|  | Cluster-10307.81775 | 5.06E-03 | 3.330591929 | up |
|  | Cluster-10307.83164 | 7.71E-11 | 5.180850163 | up |
|  | Cluster-10307.21303 | 2.49E-04 | 2.617072211 | up |
|  | Cluster-10307.40598 | 6.55E-06 | 3.008927706 | up |
|  | Cluster-10307.4971 | 8.13E-05 | 5.517407602 | up |
|  | Cluster-10307.5021 | 6.36E-10 | 8.366147047 | up |
|  | Cluster-10307.79869 | 4.86E-02 | 2.124818922 | up |
|  | Cluster-10307.2791 | 6.99E-07 | 5.455421897 | up |
|  | Cluster-10307.34524 | 1.70E-01 | 1.99958463 | up |
|  | Cluster-10307.32442 | 2.40E-06 | 3.138070957 | up |
|  | Cluster-7157.0 | 5.92E-03 | -3.558176866 | down |
| AT2 | Cluster-10307.49831 | 5.20E-06 | 1.927054282 | up |
|  | Cluster-10307.55417 | 3.36E-05 | 5.55595824 | up |
| CHS | Cluster-10307.82854 | 1.96E-02 | 2.137382746 | up |
| C4Ha | Cluster-10307.25395 | 5.78E-05 | 2.558402847 | up |
|  | Cluster-10307.4519 | 2.22E-03 | 4.572769334 | up |
|  | Cluster-10307.21895 | 1.34E-01 | -1.676459595 | down |
| HCT | Cluster-10307.83910 | 6.31E-03 | 4.621369836 | up |
| CCoAOMT | Cluster-10307.82368 | 4.26E-05 | 4.254278345 | up |
| F3H | Cluster-10307.23258 | 3.49E-02 | 1.576185258 | up |
| LAR2 | Cluster-10307.9753 | 2.33E-04 | 2.124519835 | up |
|  | Cluster-10307.79653 | 1.89E-02 | 2.49867932 | up |
|  | Cluster-10307.78333 | 1.24E-01 | -1.753436506 | down |
| GSCOC | Cluster-6365.0 | 3.90E-02 | 2.189608881 | up |
| CFOL | Cluster-10307.10288 | 3.74E-02 | -2.541402034 | down |
|  | Cluster-10307.49896 | 6.68E-02 | -1.143582386 | down |
| CHI | Cluster-10307.69071 | 6.94E-02 | -1.174201473 | down |
| DFR | Cluster-10307.30160 | 1.35E-02 | -1.961688526 | down |
| LSAT | Cluster-10307.44404 | 8.40E-02 | -2.366982942 | down |

| **Table S9. List of relativity between differential expression structural genes of flavonoid and differential accumulation flavonoid metabolites** | | | | |
| --- | --- | --- | --- | --- |
| **Gene Name** | **Gene ID** | **Index** | **Compounds** | **Correlation  coefficent** |
|  |  |  |  |  |
| CsUGT134 | Cluster-10307.5267 | Lmlp006175 | Isosalipurposide (Phlorizin Chalcone) | -0.821465886 |
|  |  | Lmjp002596 | Quercetin-3-O-sambubioside* | -0.996874832 |
|  |  | Hmcp001618 | Quercetin-3-O-(2''-O-Xylosyl)rutinoside | -0.836259553 |
|  | Cluster-10307.10035 | mws1179 | Naringenin-7-O-glucoside (Prunin) | -0.857463558 |
| DFR | Cluster-10307.30160 | mws1179 | Naringenin-7-O-glucoside (Prunin) | 0.876974935 |
|  |  | Lmjp002596 | Quercetin-3-O-sambubioside* | 0.918558127 |
| LAR2 | Cluster-10307.9753 | mws1179 | Naringenin-7-O-glucoside (Prunin) | -0.837489463 |
|  |  | Lmlp006175 | Isosalipurposide (Phlorizin Chalcone) | -0.88435483 |
|  |  | Lmjp002596 | Quercetin-3-O-sambubioside* | -0.967289185 |
|  |  | Hmcp001618 | Quercetin-3-O-(2''-O-Xylosyl)rutinoside | -0.91938102 |
| C4Ha | Cluster-10307.4519 | MWSHY0189 | Apigenin-7-O-glucoside(Cosmosiin) | -0.919072282 |
|  |  | mws1179 | Naringenin-7-O-glucoside (Prunin) | -0.942888373 |
|  |  | Lmlp006175 | Isosalipurposide (Phlorizin Chalcone) | -0.857174703 |
|  |  | Lmjp002596 | Quercetin-3-O-sambubioside* | -0.830994953 |
| CFOL | Cluster-10307.10288 | Lmlp006175 | Isosalipurposide (Phlorizin Chalcone) | 0.902000449 |
|  |  | Lmjp002596 | Quercetin-3-O-sambubioside* | 0.910565548 |
|  |  | Hmcp001618 | Quercetin-3-O-(2''-O-Xylosyl)rutinoside | 0.945820818 |
|  | Cluster-10307.49896 | mws1179 | Naringenin-7-O-glucoside (Prunin) | 0.85065959 |
|  |  | Lmlp006175 | Isosalipurposide (Phlorizin Chalcone) | 0.830004746 |
|  |  | MWSHY0080 | Luteolin-7-O-neohesperidoside (Lonicerin) | -0.834744123 |
|  |  | MWSHY0050 | Kaempferol-3-O-rutinoside(Nicotiflorin)* | -0.874751561 |
| CHI | Cluster-10307.69071 | mws1179 | Naringenin-7-O-glucoside (Prunin) | 0.811405756 |
|  |  | Lmlp006175 | Isosalipurposide (Phlorizin Chalcone) | 0.868538263 |
|  |  | Lmjp002596 | Quercetin-3-O-sambubioside* | 0.96459718 |
|  |  | Hmcp001618 | Quercetin-3-O-(2''-O-Xylosyl)rutinoside | 0.819786618 |
| LOC | Cluster-10307.11602 | Lmjp002596 | Quercetin-3-O-sambubioside* | -0.815605411 |
|  |  | Hmcp001618 | Quercetin-3-O-(2''-O-Xylosyl)rutinoside | -0.849260277 |
|  | Cluster-10307.81571 | Lmlp006175 | Isosalipurposide (Phlorizin Chalcone) | -0.830521144 |
|  |  | Lmjp002596 | Quercetin-3-O-sambubioside* | -0.991190951 |
|  |  | Hmcp001618 | Quercetin-3-O-(2''-O-Xylosyl)rutinoside | -0.874657103 |
|  | Cluster-10307.81775 | mws1179 | Naringenin-7-O-glucoside (Prunin) | -0.919532017 |
|  |  | Lmjp002596 | Quercetin-3-O-sambubioside* | -0.898044688 |
|  | Cluster-10307.21303 | mws1179 | Naringenin-7-O-glucoside (Prunin) | -0.916155147 |
|  |  | MWSHY0080 | Luteolin-7-O-neohesperidoside (Lonicerin) | 0.832039569 |
|  |  | MWSHY0050 | Kaempferol-3-O-rutinoside(Nicotiflorin)* | 0.849149109 |
|  |  | Lmjp002596 | Quercetin-3-O-sambubioside* | -0.886916962 |
|  | Cluster-10307.32442 | mws1179 | Naringenin-7-O-glucoside (Prunin) | -0.849077696 |
|  |  | Lmjp002596 | Quercetin-3-O-sambubioside* | -0.877893374 |
|  | Cluster-10307.40598 | MWSHY0189 | Apigenin-7-O-glucoside(Cosmosiin) | -0.825698961 |
|  |  | mws1179 | Naringenin-7-O-glucoside (Prunin) | -0.926268527 |
|  |  | Lmlp006175 | Isosalipurposide (Phlorizin Chalcone) | -0.836988205 |
|  |  | Lmjp002596 | Quercetin-3-O-sambubioside* | -0.95357033 |
|  | Cluster-10307.4971 | MWSHY0080 | Luteolin-7-O-neohesperidoside (Lonicerin) | 0.819795224 |
|  |  | MWSHY0050 | Kaempferol-3-O-rutinoside(Nicotiflorin)* | 0.833462753 |
|  | Cluster-10307.79869 | mws1179 | Naringenin-7-O-glucoside (Prunin) | -0.840077688 |
|  |  | Lmlp006175 | Isosalipurposide (Phlorizin Chalcone) | -0.884097743 |
|  |  | Lmjp002596 | Quercetin-3-O-sambubioside* | -0.989325427 |
|  |  | Hmcp001618 | Quercetin-3-O-(2''-O-Xylosyl)rutinoside | -0.875537894 |
|  | Cluster-10307.2791 | mws1179 | Naringenin-7-O-glucoside (Prunin) | -0.847873631 |
|  |  | Lmlp006175 | Isosalipurposide (Phlorizin Chalcone) | -0.8236496 |
|  |  | Lmjp002596 | Quercetin-3-O-sambubioside* | -0.997148403 |
|  |  | Hmcp001618 | Quercetin-3-O-(2''-O-Xylosyl)rutinoside | -0.815126575 |
| GSCOC | Cluster-6365.0 | mws1179 | Naringenin-7-O-glucoside (Prunin) | -0.887148492 |
|  |  | Lmlp006175 | Isosalipurposide (Phlorizin Chalcone) | -0.941123088 |
|  |  | Lmjp002596 | Quercetin-3-O-sambubioside* | -0.956670081 |
|  |  | Hmcp001618 | Quercetin-3-O-(2''-O-Xylosyl)rutinoside | -0.824837232 |
| CCoAOMT | Cluster-10307.82368 | Lmlp006175 | Isosalipurposide (Phlorizin Chalcone) | -0.848762541 |
|  |  | Lmjp002596 | Quercetin-3-O-sambubioside* | -0.920764139 |
|  |  | Hmcp001618 | Quercetin-3-O-(2''-O-Xylosyl)rutinoside | -0.817958873 |
| AT2 | Cluster-10307.49831 | mws1179 | Naringenin-7-O-glucoside (Prunin) | -0.898255398 |
|  |  | Lmlp006175 | Isosalipurposide (Phlorizin Chalcone) | -0.94678871 |
|  |  | MWSHY0080 | Luteolin-7-O-neohesperidoside (Lonicerin) | 0.81175841 |
|  |  | MWSHY0050 | Kaempferol-3-O-rutinoside(Nicotiflorin)* | 0.846579319 |
|  |  | Lmjp002596 | Quercetin-3-O-sambubioside* | -0.954909876 |
|  |  | Hmcp001618 | Quercetin-3-O-(2''-O-Xylosyl)rutinoside | -0.895789995 |
|  | Cluster-10307.55417 | MWSHY0189 | Apigenin-7-O-glucoside(Cosmosiin) | -0.864850539 |
|  |  | mws1179 | Naringenin-7-O-glucoside (Prunin) | -0.939367429 |
|  |  | Lmlp006175 | Isosalipurposide (Phlorizin Chalcone) | -0.853841879 |
|  |  | Lmjp002596 | Quercetin-3-O-sambubioside* | -0.8870022 |

**Table S10 qRT PCR of Relative gene expression between differential expression structural genes of flavonoid and differential accumulation flavonoid metabolites**

|  | Genes ID | Compounds | Class II |
| --- | --- | --- | --- |
| 1 | Cluster-10307.30160 | Naringenin-7-O-glucoside (Prunin) | Flavanones |
| 2 | Cluster-10307.4971 | Naringenin-7-O-glucoside (Prunin) | Flavanones |
| 3 | Cluster-10307.4519 | Naringenin-7-O-glucoside (Prunin) | Flavanones |
|  |  | Isosalipurposide (Phlorizin Chalcone) | Chalcones |
| 4 | Cluster-6365.0 | Naringenin-7-O-glucoside (Prunin) | Flavanones |
|  |  | Isosalipurposide (Phlorizin Chalcone) | Chalcones |
| 5 | Cluster-10307.5267 | Naringenin-7-O-glucoside (Prunin) | Flavanones |
|  |  | Isosalipurposide (Phlorizin Chalcone) | Chalcones |
|  |  | Quercetin-3-O-(2''-O-Xylosyl)rutinoside | Flavonols |
| 6 | Cluster-10307.9753 | Naringenin-7-O-glucoside (Prunin) | Flavanones |
|  |  | Isosalipurposide (Phlorizin Chalcone) | Chalcones |
| 7 | Cluster-10307.69071 | Isosalipurposide (Phlorizin Chalcone) | Chalcones |
| 8 | Cluster-10307.82368 | Isosalipurposide (Phlorizin Chalcone) | Chalcones |
| 9 | Cluster-10307.10288 | Isosalipurposide (Phlorizin Chalcone) | Chalcones |
| 10 | Cluster-10307.81571 | Isosalipurposide (Phlorizin Chalcone) | Chalcones |
| 11 | Cluster-10307.55417 | Apigenin-7-O-glucoside(Cosmosiin) | Flavones |
|  |  | Quercetin-3-O-sambubioside | Flavonols |
